# Modulation of A_1_ and A_2B_ adenosine receptor activity: a new strategy to sensitise glioblastoma stem cells to chemotherapy

**DOI:** 10.1038/cddis.2014.487

**Published:** 2014-11-27

**Authors:** S Daniele, E Zappelli, L Natali, C Martini, M L Trincavelli

**Affiliations:** 1 Department of Pharmacy, University of Pisa, Pisa, Italy

## Abstract

Therapies that target the signal transduction and biological characteristics of cancer stem cells (CSCs) are innovative strategies that are used in combination with conventional chemotherapy and radiotherapy to effectively reduce the recurrence and significantly improve the treatment of glioblastoma multiforme (GBM). The two main strategies that are currently being exploited to eradicate CSCs are (a) chemotherapeutic regimens that specifically drive CSCs toward cell death and (b) those that promote the differentiation of CSCs, thereby depleting the tumour reservoir. Extracellular purines, particularly adenosine triphosphate, have been implicated in the regulation of CSC formation, but currently, no data on the role of adenosine and its receptors in the biological processes of CSCs are available. In this study, we investigated the role of adenosine receptor (AR) subtypes in the survival and differentiation of CSCs isolated from human GBM cells. Stimulation of A_1_AR and A_2B_AR had a prominent anti-proliferative/pro-apoptotic effect on the CSCs. Notably, an A_1_AR agonist also promoted the differentiation of CSCs toward a glial phenotype. The differential effects of the two AR agonists on the survival and/or differentiation of CSCs may be ascribed to their distinct regulation of the kinetics of ERK/AKT phosphorylation and the expression of hypoxia-inducible factors. Most importantly, the AR agonists sensitised CSCs to the genotoxic activity of temozolomide (TMZ) and prolonged its effects, most likely through different mechanisms, are as follows: (i) by A_2B_AR potentiating the pro-apoptotic effects of TMZ and (ii) by A_1_AR driving cells toward a differentiated phenotype that is more sensitive to TMZ. Taken together, the results of this study suggested that the purinergic system is a novel target for a stem cell-oriented therapy that could reduce the recurrence of GBM and improve the survival rate of GBM patients.

Glioblastoma multiforme (GBM), classified as grade IV on the World Health Organization scale,^1^ is the most common type of primary malignant brain tumour.^2^ The current therapeutic strategy includes surgery followed by radiation and chemotherapy using temozolomide (TMZ). This therapeutic approach slightly improves the survival rate of GBM patients, but their prognosis remains poor and most patients die of tumour recurrence.^3^ The causes of the recurrence of GBM are complex and include the high proliferative index of the tumour cells and their resistance to chemotherapy and radiotherapy, particularly in the case of the cancer stem cells (CSCs). These cells have been proposed to not only initiate the genesis of GBM and contribute to its highly proliferative nature, but to also be the basis for its recurrences following treatment. Moreover, it has been reported that the most aggressive or refractory cancers contain the highest number of CSCs.^4, 5, 6^

These findings suggest that innovative stem cell-orientated therapy may be an effective strategy to reduce tumour recurrence and significantly improve GBM treatment outcomes.^7, 8, 9, 10, 11, 12, 13, 14, 15, 16, 17, 18^ This type of therapy may not be easy to implement because CSCs have been shown to have a low level of reactive oxygen species^19^ and to be more resistant to ionising radiation,^20^ vincristine,^21^ hypoxia and other chemotherapeutics^22^ compared with non-CSCs. In contrast, the preferential elimination of the CSC population may contribute to the effectiveness of TMZ, which is the most effective pharmacologic agent used in glioma treatment;^23^ however, the activity of TMZ appears to be short lived because the drug causes the reversible blockage of the cell cycle of CSCs.^24^ Moreover, long-term TMZ therapy results in the occurrence of drug-resistant GBM cells,^25^ indicating the need to develop distinct strategies to overcome this resistance.

Extracellular purines have been implicated in several aspects of GBM biology, such as proliferation,^26^ migration,^27^ invasion^28^ and death.^29^ The concentration of adenosine in the extracellular fluid of glioma tissue was reported to be in the low micromolar range,^30^ which is sufficiently high to stimulate all the four of the adenosine receptor (AR) subtypes (A_1_, A_2A_, A_2B_ and A_3_).^31^ Each of the ARs have a pivotal role in the control of tumour growth and invasiveness^32, 33, 34^ but to date, no data on their role in CSC biology are available. Recently, it was demonstrated that treatment with adenosine triphosphate reduced the rate of sphere formation by glioma cells and that purinergic receptors are differentially expressed in spheres of tumour cells and adherent cells.^33^ In this study, we investigated the role of AR subtypes in the survival and differentiation of CSCs. Globally, our data clarified the role of each AR subtype in CSC functionality and suggested that the purinergic system is a novel pharmacological target for the development of new anti-CSC therapies, particularly those aimed at the treatment of GBM recurrences.

## Results

### Isolation of the tumour stem cell populations

The formation of neurospheres *in vitro* in U87MG and U343MG cell cultures was induced by using specific neural stem cell (NSC) medium^35^ (Supplementary Figure 1A). The spheres obtained using either U87MG and U343MG cells included significantly more CD133/nestin^+^ cells and a smaller percentage of GFAP^+^ cells compared with the pool of whole GBM cells (Supplementary Figures 1B, C and D).

#### Expression and functionality of the ARs in GBM cells and CSCs

**Figure 1:**
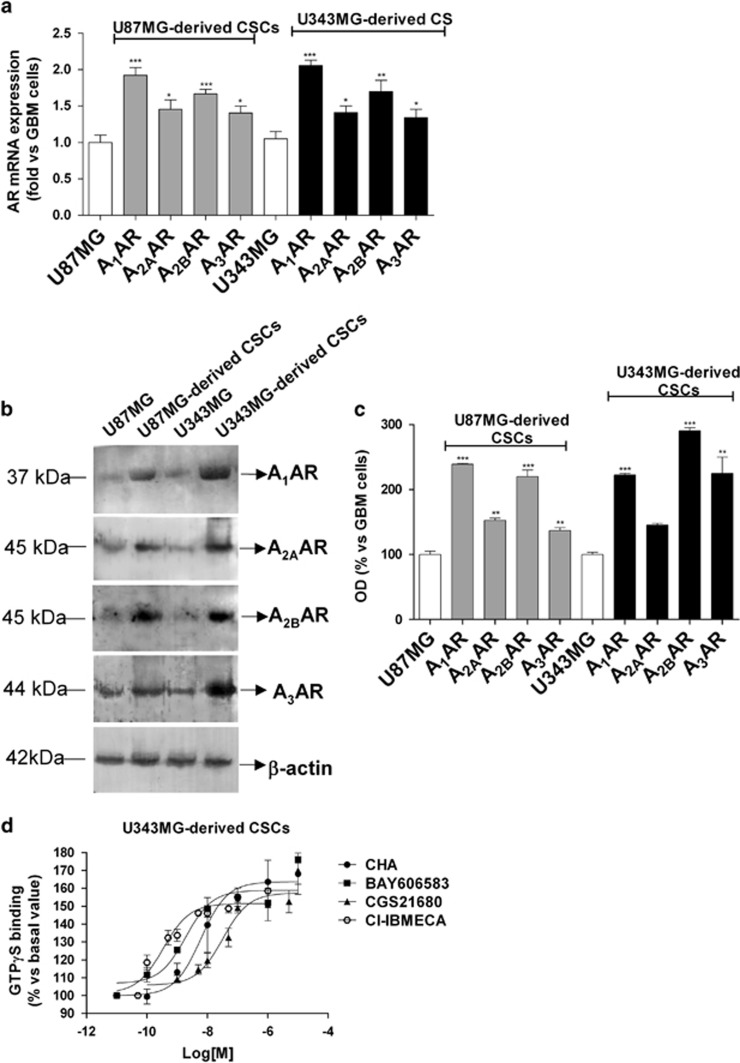
Expression of ARs in CSCs derived from U87MG and U343MG cells. (**a**) The total RNA was extracted from whole U87MG cells and U343MG cells and from the respectively derived CSCs. The relative quantification of the AR subtype mRNAs was performed using real-time RT-PCR, as described in the Materials and methods section. The data were expressed as the fold change relative to the level of expression of each AR subtype in GBM cells (each set to 1) and they are the mean values±S.E.M. of three different experiments. (**b** and **c**) Cell lysates were prepared from whole U87MG cells, U343MG cells and the CSCs derived from them. The AR protein levels were evaluated using western blot analysis, using *β*-actin as the loading control. (**b**) Representative western blots. (**c**) Densitometric analysis of the immunoreactive bands performed using the ImageJ program. The data were expressed as the percentage relative to the level of expression in whole GBM cells, and they are the mean values±S.E.M. of three different experiments. The significance of the differences was evaluated using a one-way ANOVA with the Bonferroni post-test: *P<0.05, **P<0.01, ***P<0.001 *versus* the levels of expression in whole GBM cells. (**d**) [^35^S]GTP*γ*S binding of U343MG-derived CSCs. Membrane aliquots (30 *μ*g) obtained from CSCs were incubated with the indicated selective AR agonists at various concentrations. All of the data were expressed as the percentages of the level of basal [^35^S]GTP*γ*S binding (set to 100%), and they are the mean values±S.E.M. of three different experiments, each performed in duplicate

The expression profiles of the ARs in the GBM cell lines and in their CSCs were compared. Real-time PCR (Figure 1a) and western blot (Figures 1b and c) analyses revealed that U87MG and U343MG cells expressed all four of the AR subtypes, as previously reported for other glioma cell lines.^36, 37^ The levels of expression of ARs were found to be further increased in CSC-derived neurospheres compared with those of whole GBM cells (Figure 1).

The functional responsiveness of the CSC ARs was evaluated using the GTP*γ*S-binding assay.^38^ As shown in Figure 1d, treatment with CHA and BAY606583 increased the level of GTP*γ*S binding in a concentration-dependent manner, yielding EC_50_ values of 6.1±0.5 and 1.9±0.2 nM, respectively; these values are comparable to their affinity constant values vis a vis A_1_AR and A_2B_AR.^39, 40^ In a similar manner, CGS21680 and Cl-IBMECA stimulated GTP*γ*S binding, yielding affinity constant values of 41.9±4.5 and 0.46±0.06 nM, respectively, consistent with those previously reported.^41, 42^

#### Effect of AR ligands on CSC proliferation/viability

Incubating U87MG or U343MG cells with the selective AR agonists for 24 or 48 h did not induce any significant change in the cell proliferation rate (Supplementary Figure 2). After 4 days of A_1_AR, A_2B_AR or A_3_AR stimulation, the rate of cell proliferation was enhanced (Supplementary Figures 2A and B), consistent with published data.^31^

**Figure 2:**
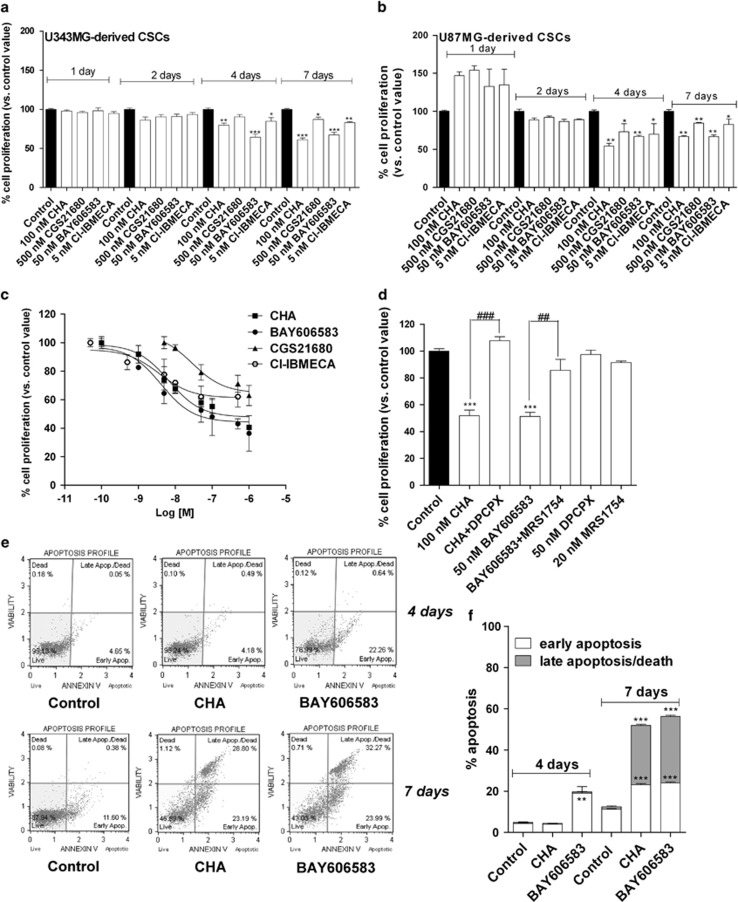
Effects of AR agonists on CSC proliferation. U343MG-derived CSCs (**a**) or U87MG-derived CSCs (**b**) were treated for the indicated number of days with the A_1_AR agonist (CHA), the A_2A_AR agonist (CGS21680), the A_2B_AR agonist (BAY606583) or the A_3_AR agonist Cl-IBMECA at selected concentrations (corresponding to tenfold the values of the affinity constants). (**c**) CSCs derived from U343MG cells were incubated for 7 days with the AR agonists at increasing concentrations. (**d**) CSCs derived from U343MG cells were incubated for 7 days with 100 nM CHA, in the absence or in the presence of the A_1_AR antagonist DPCPX (50 nM), or with 50 nM BAY606583, in the absence or in the presence of the A_2B_AR antagonist MRS1754 (20 nM). At the end of the treatments, cell proliferation was evaluated using the MTS assay. The data were expressed as a percentage with respect to that of untreated cells (control), which was set to 100%, and they are the mean values±S.E.M. of three independent experiments, each performed in duplicate. (**e** and **f**) CSCs were treated for 4 or 7 days with NSC medium containing DMSO (control), 100 nM CHA or 50 nM BAY606583. At the end of treatments, the cells were collected and the degree of phosphatidylserine externalisation was evaluated using the Annexin V protocol, as described in the Materials and method section. (**f**) The data were expressed as the percentage of apoptotic cells (early-stage apoptotic cells shown in white, late-stage apoptotic/necrotic cells shown in grey) relative to the total number of cells. The data shown are the mean values±S.E.M. of three different experiments. The significance of the differences was determined using a one-way ANOVA with the Bonferroni post-test. *P<0.05, **P<0.01, ***P<0.001 *versus* control. ^##^*P*<0.01, ^###^*P*<0.001 *versus* agonist alone

Similar experiments were then performed on CSCs: none of AR agonists had a significant effect on the growth of the neurospheres after 24 or 48 h of treatment (Figures 2a and b). In contrast, when the neurospheres were treated for 4 or 7 days with each of the AR ligands, a significant decrease in cell proliferation was observed (Figures 2a and b). Cell counting showed that all of the AR ligands significantly reduced the percentage of living cells beginning at 4 days of treatment, with a greater effect evident after 7 days of treatment (Supplementary Figure 3). These data suggested that the reduced rate of CSC proliferation can be ascribed, at least partially, to a reduction in the number of living cells.

As similar results on cellular viability were obtained in CSCs isolated from U87MG and U343MG cell lines, the subsequent experiments were focused only on U343MG-derived CSCs, chosen as representative GBM cell line.

Then, a dose-response study of the AR agonists was performed. CHA and BAY606583 induced a concentration-dependent inhibition of CSC proliferation, with a maximal percentage of CSC growth inhibition of 59.9±1.5 and 63.6±7.2, respectively, and IC_50_ values of 11.2±1.0 and 3.47±0.39 nM, respectively (Figure 2c), the latter being values that are comparable to the degree of affinity of these ligands for the A_1_AR or A_2B_AR.^39, 40^ In contrast, CGS21680 and Cl-IBMECA had moderate growth-inhibitory effects, with a maximal percentage of CSC growth inhibition of 37.0±3.5 and 37.9±4.2, respectively (Figure 2c). The effects of the A_1_AR and A_2B_AR agonists appeared to be completely counteracted by the selective AR antagonists, DPCPX and MRS1754 (Figure 2d), indicating that the anti-proliferative effects of CHA and BAY606583 on CSCs are specifically mediated by the activation of A_1_AR and A_2B_AR, respectively.

We then investigated whether the reduction in cell proliferation elicited by the AR agonists could be associated with cellular apoptosis. Treating the cells for 4 days with BAY606583 induced a slight but significant early apoptosis (Figure 2e). In contrast, although a reduction in cell proliferation was demonstrated using the MTS assay (Figure 2d), CHA did not induce cellular apoptosis at this point of treatment (Figures 2e and f). After 7 days, both CHA and BAY606583 induced a significant level of phosphatidylserine externalisation, both without (early apoptosis) and with the occurrence of 7-amino-actinomysin 11 binding to DNA (late apoptosis/death) (Figures 2e and f), with a total percentage of cellular apoptosis of 52.0±0.6 and 56.3±2.6, respectively. These data are consistent with the reduction in the percentage of living cells revealed using the Trypan blue exclusion assay (Supplementary Figure 3). Treatment with CGS21680 and Cl-IBMECA significantly induced early stage of CSC apoptosis, with total percentages of apoptotic cells of 18.8±1.5 and 19.4±1.8, respectively (Supplementary Figure 4), confirming that stimulating A_2__A_AR and A_3_ AR had only moderate effects on CSC viability/apoptosis.

#### Effects of A_1_AR and A_2B_AR agonists on the morphology and differentiation of CSCs

As the most promising anti-proliferative effects of the AR agonists were obtained using CHA and BAY606583, the roles of the A_1_AR and A_2B_AR subtypes in CSCs were further investigated. The effects of selective A_1_ or A_2B_AR agonists on CSC morphology were thus evaluated.

**Figure 3:**
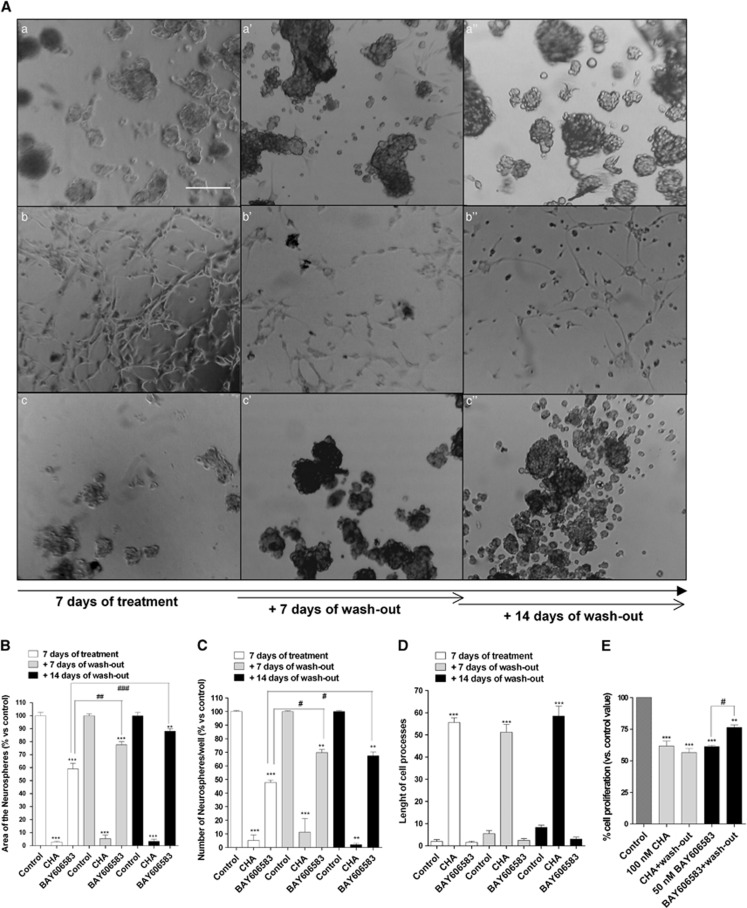
Effect of A_1_AR and A_2B_AR agonists on the sphere-derived cell morphology of the CSC-derived cellular spheres. CSCs were treated for 7 days with complete NSC medium containing DMSO (control, a), 100 nM CHA (b) or 50 nM BAY606583 (c). At the end of treatment periods, the drug-containing media were replaced with fresh drug-free NSC medium, and cells were cultured for another 7 (a', b', c') or 14 days (a”, b”, c”). (**A**) Representative micrographs taken after 7 days of treatment and after 7 or 14 days of drug wash-out are shown. The area of the culture plates occupied by the spheres (**B**), the number of spheres (**C**) and the length of the cellular processes (**D**) were scored after 7 days of treatment and after 7 and 14 days of drug wash-out. The counts represent the mean values±S.E.M. of three independent experiments. (**E**) CSCs were treated as in **A**, and cell proliferation was evaluated using the MTS assay. The data were expressed as percentages relative to that of the untreated cells (control), which was set at 100%, and they are the mean values±S.E.M. of three independent experiments, each performed in duplicate. The significance of the differences was determined using a one-way ANOVA with the Bonferroni post-test: **P<0.01, ***P<0.001 *versus* control; ^#^*P*<0.05, ^##^*P*<0.01, ^###^*P*<0.001 *versus* cells treated for seven days

After 7 days of treatment, BAY606583 caused a significant reduction in the area occupied by the cells and the number of neurospheres (Figure 3). These effects were observed after 4 days of treatment, although to a lesser extent (Supplementary Figure 5).

When CSCs were incubated with CHA for 4 days, an increase in the number of adherent cells was observed, and the CSCs began to exhibit cellular processes (Supplementary Figure 5). After 7 days of treatment, an almost loss of floating spheres was noticed (Figures 3a and d) and the cells exhibited significant outgrowth of processes. Comparing these data with those obtained in the Annexin V-staining assay suggested that CHA first induced CSC differentiation and, in a later phase, triggered the apoptosis of the differentiated cells.

The receptor specificity of the CHA- and BAY606583-mediated effects was confirmed using the selective antagonists DPCPX and MRS1754 (Supplementary Figure 6).

**Figure 4:**
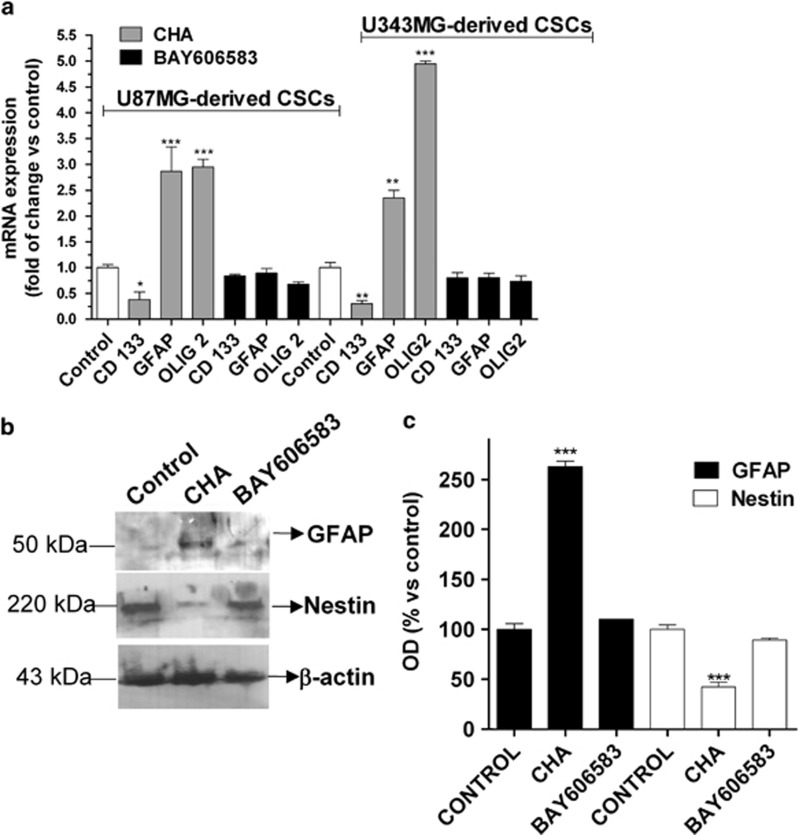
Effects of A_1_AR and A_2B_AR agonists on the expression of differentiation and stemness markers in CSCs. (**a**) CSCs derived from U87MG or U343MG cells were treated for 7 days with NSC medium containing DMSO (control), 100 nM CHA or 50 nM BAY606583. At the end of treatment periods, the total RNA was extracted, and relative quantification of the mRNAs for the stem cell marker CD133, the astrocyte marker GFAP, and the oligodendrocyte marker Olig2 were performed using RT-PCR, as described in the Materials and methods section. The data were expressed as the fold change *versus* the levels of the control and they are the mean values±S.E.M. of three different experiments. (**b** and **c**) CSCs from U343MG cells were treated as in **a**; at the end of treatment periods, cell lysates were subjected to western blot analysis using antibodies specific for the stem cell marker nestin or the glial-cell marker GFAP. (**b**) Representative western blots. (**c**) The bar graph shows the results of the quantitative analysis of the western blots, which was performed using the ImageJ program. The data were expressed as the percentage of optical density of the immunoreactive band relative to that of the control, set to 100% and are the mean values±S.E.M. of three different experiments. The significance of the differences was determined using a one-way ANOVA with the Bonferroni post-test: *P<0.05, **P<0.01, ***P<0.001 *versus* control

To further investigate the mechanisms through which CHA and BAY606583 affected CSC morphology, we then assessed the expression of stem cell and differentiation markers in CSCs upon their stimulation with these agonists. Real-time PCR (Figure 4a) and western blot analysis (Figures 4b and c) revealed that BAY606583 did not significantly affect the levels of expression of stem cell markers and of the astrocyte marker, suggesting that stimulation of A_2B_AR did not change the CSC phenotype. In comparative studies, no alteration of the CSC phenotype was observed with either CGS21680 or Cl-IBMECA treatment (Supplementary Figure 7). In contrast, in CHA-treated CSCs, a significant decrease in the expression of the stemness markers (Figure 4) and a significant increase in the expression of GFAP and Olig2 was observed, demonstrating that CHA promoted CSC differentiation toward a glial phenotype.

Next, we assessed whether CHA- or BAY606583-treated cells could resume growing after the removal of the drug. After the BAY606583 challenge and a wash-out period of 7 or 14 days, spheres began to resume proliferation (Figures 3a–c). In contrast, the CHA-treated cells retained their differentiated morphology even after 14 days of drug wash-out, although some floating spheres were observed (Figures 3a–d). These results were confirmed using the MTS assay (Figure 3e).

#### Effects of the A_1_AR and A_2B_AR agonists on pro-apoptotic/differentiating pathways

**Figure 5:**
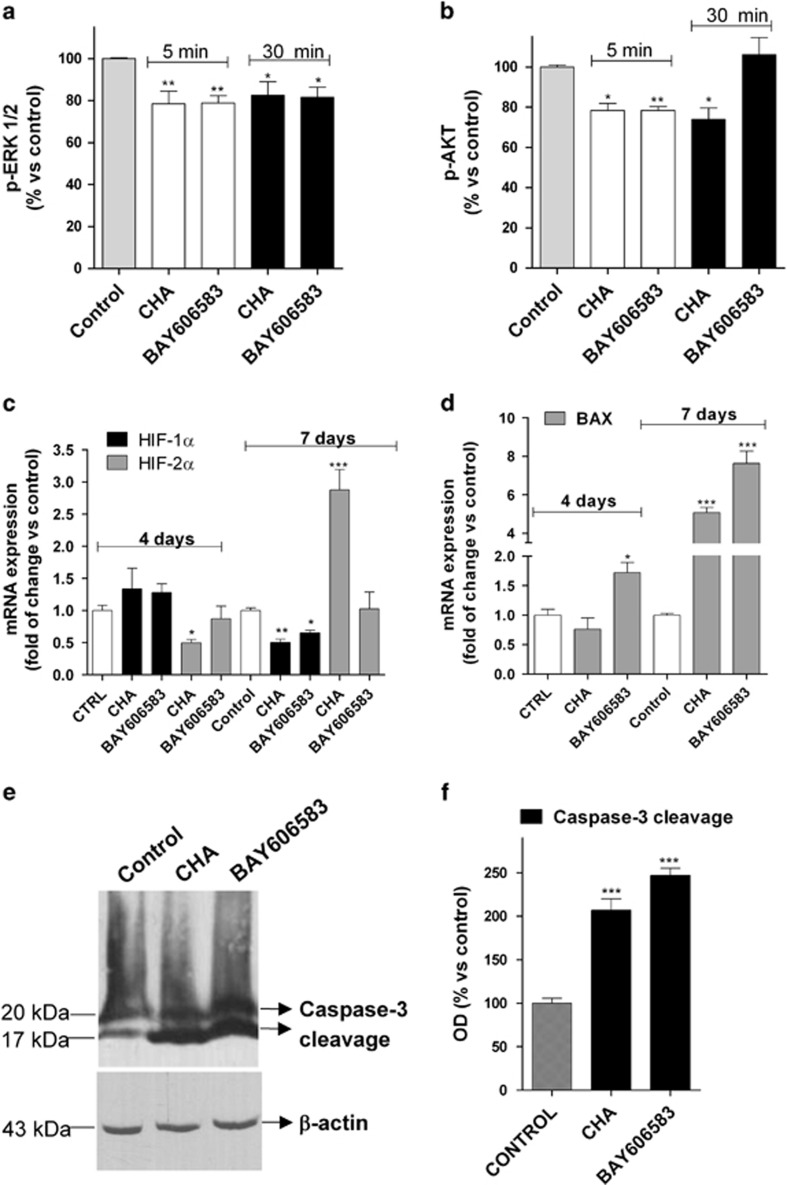
Effects of A_1_AR and A_2B_AR agonists on pro-apoptotic/differentiating pathways. (**a** and **b**) U343MG-derived CSCs were treated for 5 or 30 min with complete medium containing DMSO (control), 100 nM CHA or 50 nM BAY606583. Following the treatments, the levels of ERK 1/2 (**a**) and AKT (**b**) phosphorylation were evaluated using ELISA kits, as described in the Materials and methods section. The data were expressed as the percentage of phosphorylated AKT or ERK1/2 relative to those of untreated cells (control), which were set at 100%, and are the mean values±S.E.M. of three independent experiments performed in triplicate. (**c** and **d**) CSCs derived from U343MG cells were treated for 4 or 7 days with NSC medium containing DMSO (control), 100 nM CHA or 50 nM BAY606583. At the end of the treatment periods, the total RNA was extracted, and the relative quantification of the HIFs (**c**) and Bax (**d**) mRNAs was performed using RT-PCR. The data were expressed as the fold change *versus* the level of the control and are the mean values±S.E.M. of three different experiments. (**e** and **f**) CSCs derived from U343MG cells were treated as in **a** for 7 days. The cell lysates were subjected to western blot analysis using an antibody specific for caspase-3 cleavage products. (**e**) Representative western blots. (**f**) The bar graph shows the results of the quantitative analysis of the western blots, which was performed using the ImageJ program. The data were expressed as the percentage of optical density of the immunoreactive band relative to that of the control, set to 100%, and are the mean values±S.E.M. of two different experiments. The significance of the differences was determined using a one-way ANOVA with the Bonferroni post-test: *P<0.05, **P<0.01, ***P<0.001 *versus* control

Different signalling pathways have been demonstrated to have a pivotal role in CSC self-renewal, migration and differentiation,^43^ including the phosphatidylinositol 3-kinase (PI3K)/AKT and the MEK/ERK pathways.^44^ Thus, the effects of the A_1_AR and A_2B_AR agonists on the levels of total and phosphorylated ERK1/2 and AKT were first investigated. CSC incubation with AR agonists did not alter total ERK or AKT levels (Supplementary Figure 8). Both CHA and BAY606583 induced a modest but significant inhibition of ERK1/2 phosphorylation, which persisted for the 30 min of CSC incubation (Figure 5a). Both ligands also affected the p-AKT level, although with a different kinetic pattern; whereas CHA inhibited AKT phosphorylation for up to 30 min, BAY606583 caused only a transient blockage of AKT phosphorylation (Figure 5b), suggesting that the kinetics of AKT inhibition may explain the different effects of the two AR agonists.

Several studies have demonstrated that hypoxia-inducible factors (HIFs), including HIF-1*α* and HIF-2*α*, have potential biological effects on maintaining the stemness of these cells.^45, 46, 47, 48, 49^ Therefore, the modulation of HIF transcriptional activity upon incubation with the A_1_ or A_2B_AR agonists was investigated. Neither of ligands altered the HIF-1*α* level after 4 days of treatment, whereas both of them caused a significant reduction in the HIF-1*α* level after 7 days of treatment (Figure 5c), consistent with the marked inhibition of CSC proliferation at this time point.^50, 51, 52^

BAY606583 treatment did not affect the level of HIF-2*α* mRNA at the analysed time points; in contrast, CHA caused a slight but significant reduction in the level of HIF-2*α* mRNA by 4 days of treatment (Figure 5c), consistent with the reduction in the levels of expression of the stem cell markers caused by its differentiation-promoting effects. Interestingly, 7 days of CHA treatment enhanced the HIF-2*α* transcriptional activity, indicating that a different HIF-2*α*-mediated mechanism may be in effect in CHA-differentiated CSCs. We then investigated whether the AR ligands modulated the expression of the apoptosis regulator Bax, a member of Bcl-2 protein family,^53^ and of the executioner protein caspase-3, responsible for biochemical and morphological hallmarks of apoptosis.^54^

BAY606583 induced a significant enhancement of the Bax mRNA level starting at 4 days of treatment, with a maximal effect at the 7th day (Figure 5c). In contrast, CHA increased Bax transcriptional activity only after 7 days of treatment (Figure 5d). At this latter time point, both agonists had also induced a significant level of caspase-3 cleavage (Figures 5e and f).

#### Combined effect of the A_1_/A_2B_AR ligands and TMZ on CSCs

**Figure 6:**
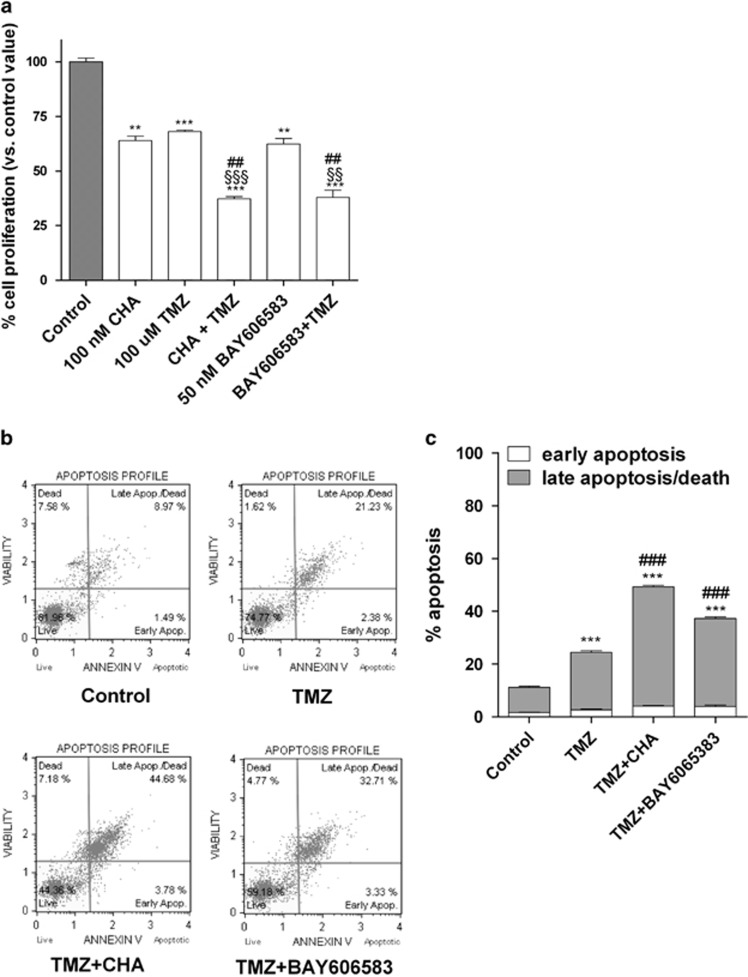
Effect of combined treatments with TMZ and an A_1_AR or A_2B_AR agonist on the proliferation and apoptosis of CSCs. (**a**) U343MG-derived CSCs were incubated for 7 days with 100 nM CHA, 50 nM BAY606583 and 100 *μ*M TMZ, alone or in combination. At the end of the treatment periods, cell proliferation was evaluated using the MTS assay. The data were expressed as percentages relative to that of the untreated cells (control), which was set at 100%, and the data shown are the mean values±S.E.M. of three independent experiments, each performed in duplicate. The significance of the differences was determined using a one-way ANOVA with the Bonferroni post-test: **P<0.01, ***P<0.001 *versus* control; ^##^*P*<0.01 *versus* AR agonist alone; ^§§^*P*<0.01, ^§§§^*P*<0.001 *versus* TMZ alone. (**b** and **c**) U343MG-derived CSCs were treated as in **a**. At the end of the treatment periods, the cells were collected and the level of phosphatidylserine externalisation was evaluated using the Annexin V-staining protocol, as described in the Materials and methods section. (**c**) The data were expressed as the percentage of apoptotic cells (data for the early-stage apoptotic cells shown in white and data for the late-stage apoptotic/necrotic cells shown in grey) *versus* the total number of cells. The data shown are the mean values±S.E.M. of three different experiments. The significance of the differences was determined using a one-way ANOVA with the Bonferroni post-test: ***P<0.001 *versus* control; ^###^*P*<0.001 *versus* TMZ alone

Considering that TMZ is the most effective pharmacologic agent used in glioma treatment and has partial and reversible effects on CSCs,^25, 55^ the effect of a combined treatment with TMZ and the A_1_AR or A_2B_AR ligand on the survival of CSCs was then examined. TMZ alone significantly inhibited CSC proliferation (Figure 6a), as previously demonstrated.^24^ Treatment for 7 days with TMZ plus CHA or with TMZ plus BAY606583 had a synergic/additive effect on the reduction of neurosphere growth (Figure 6a), most likely due to a reduction in the level of cell viability (Supplementary Figure 9).

Consistent with these data, when the alkylating agent was applied in combination with CHA or BAY606583, a significant enhancement of the level of late-stage cellular apoptosis was observed (Figures 6b and c).

**Figure 7:**
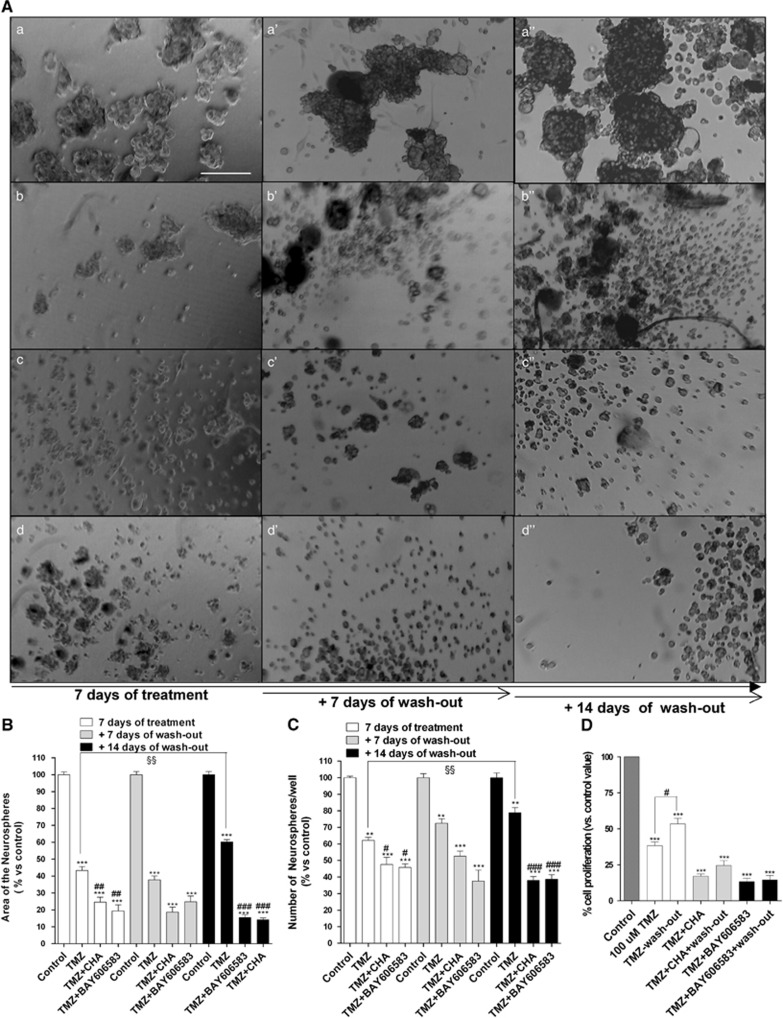
Effect of combined treatments with TMZ and A_1_AR or A_2B_AR agonists on CSC morphology. (**A**) CSCs were treated for 7 days with complete NSC medium containing DMSO (a), 100 *μ*M TMZ (b) or TMZ in combination with 100 nM CHA (c), or containing TMZ in combination with 50 nM BAY606583 (d). At the end of treatment periods, the drug-containing media were replaced with fresh drug-free NSC medium, and the cells were cultured for another 7 (a', b', c', d') or 14 (a”, b”, c”, d”) days. Representative micrographs taken after 7 days of treatment and after 7 or 14 days of drug wash-out are shown **A**. The area of the culture plates occupied by the spheres (**B**) and the number of spheres (**C**) were determined after 7 days of treatment and after 7 and 14 days of drug wash-out. The counts are the mean values±S.E.M. of three independent experiments. The significance of the differences was determined using a one-way ANOVA with the Bonferroni post-test: **P<0.05, ***P<0.001 *versus* control; ^#^*P*<0.05, ^##^*P*<0.01, ^###^*P*<0.001 *versus* TMZ alone. (**D**) CSCs were treated as in **A** and their proliferation was evaluated using the MTS assay. The data were expressed as the percentage relative to that of the untreated cells (control), which was set to 100%, and they are the mean values±S.E.M. of three independent experiments, each performed in duplicate. The significance of the differences was determined using a one-way ANOVA with the Bonferroni post-test: ***P<0.001 *versus* control; ^§§^*P*<0.01 *versus* cells treated for seven days

To further investigate the effects of such combined therapies, a morphological analysis was performed. TMZ alone significantly decreased both the area occupied by and the number of neurospheres present at 7 days (Figure 7). However, after 14 days of drug wash-out, the TMZ-treated cells recovered their normal cell growth, as confirmed by the MTS assay (Figure 7d).

When combined, TMZ and BAY606583 exerted a synergic/additive effect on inhibiting the proliferation of CSCs and significantly decreased the ability of the cells to resume proliferation (Figure 7). Interestingly, in the presence of TMZ, CHA did not induce CSC differentiation but rather had a synergic/additive effect on reducing the rate of cell proliferation relative to that of TMZ-treated cells (Figure 7). Moreover, a significant decrease in the ability of the cells to resume growth was noticed (Figures 7a–d).

**Figure 8:**
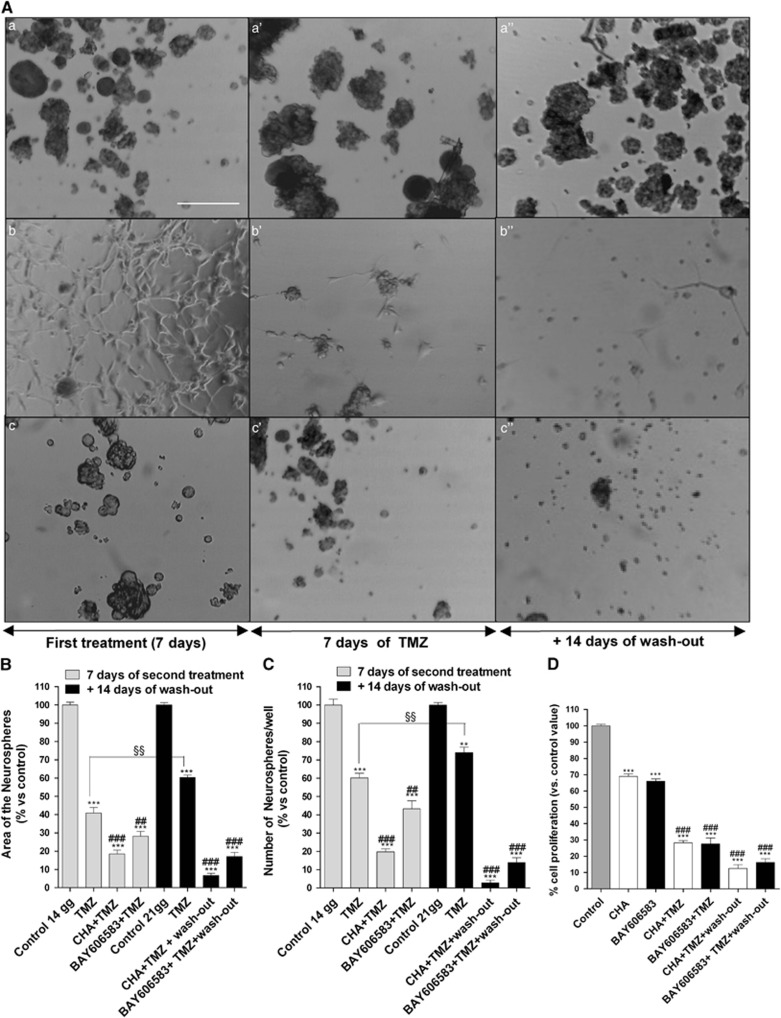
Effect of the sequential treatment of CSCs with an A_1_AR or A_2B_AR agonist and TMZ on their morphology. (**A**) CSCs were treated for 7 days with complete NSC medium containing DMSO (control) (a), 100 nM CHA (b) or 50 nM BAY606583 (c); after 7 days, the cells were treated for another 7 days with complete NSC medium containing DMSO (a') or 100 *μ*M TMZ (b', c'). At the end of treatment periods, the drug-containing media were replaced with fresh drug-free NSC medium, and the cells were cultured for another 14 days (a”, b”, c”). Representative micrographs taken after 7 days of treatment and after 7 or 14 days of drug wash-out are shown **A**. The area of the culture plates occupied by the spheres (**B**) and the number of spheres (**C**) were determined after 7 days of treatment and after 7 and 14 days of drug wash-out. The counts are the mean values±S.E.M. of three independent experiments. The significance of the differences was determined using a one-way ANOVA with the Bonferroni post-test: **P<0.01, ***P<0.001 *versus* control; ^##^*P*<0.01, ^###^*P*<0.001 *versus* TMZ alone. (**D**) CSCs were treated as in **A** and their proliferation was evaluated using the MTS assay. The data were expressed as the percentages relative to that of the untreated cells (control), which was set at 100%, and they are the mean values±S.E.M. of three independent experiments, each performed in duplicate. The significance of the differences was determined using a one-way ANOVA with the Bonferroni post-test: ***P<0.001 *versus* control; ^###^*P*<0.001 *versus* single-agent-treated cells. ^§§^*P*<0.01 *versus* cells treated for seven days with TMZ

The effects of the sequential treatment of CSCs with an AR agonist and TMZ were also investigated. A prior challenge with BAY606583 enhanced the chemotherapeutic effect of TMZ (Figure 8): the percentage of reduction of both the area occupied by and the number of CSC-derived neurospheres was significantly higher in the BAY606583–TMZ-treated cells than in the cells treated with TMZ alone (% of reduction of the area occupied by the neurospheres: TMZ alone=57.3±4.3% BAY606583–TMZ=70.2±0.6% *P*<0.001). Moreover, the ability of the BAY606583–TMZ-treated cells to resume growth at 14 days of wash-out was significantly lower than that of TMZ-treated cells (% of reduction of the area occupied by the neurospheres: TMZ alone=36.1±3.4% BAY606583–TMZ=82.6±4.1% *P*<0.001). These data were confirmed by the MTS assay (Figure 8d).

In a similar way, after CHA-induced CSC differentiation occurred, the effect of TMZ on cell proliferation was significantly stronger (Figures 8a–d). Notably, when TMZ was applied to cells that had been induced to differentiate by CHA treatment, the number of cells per well was decreased and was further decreased after 14 days of drug wash-out (CHA: 290±17; CHA-TMZ: 220±17; CHA-TMZ+wash-out: 141±14; also see Figures 7a–d). These results suggested that the CHA-differentiated cells had become sensitive to TMZ and that the effect of the sequential treatments were long lasting.

## Discussion

In this study, we found that purinergic receptors for adenosine, and particularly the A_1_AR and A_2B_AR subtypes, had a pivotal role in the survival and/or differentiation of glioblastoma CSCs. Stimulation of either the A_1_AR and A_2B_AR had a prominent anti-proliferative effect on the CSCs, and, most importantly, sensitised these cells to the chemotherapeutic activity of TMZ, are as follows: (i) by exerting synergic/addictive effects in promoting cell apoptosis and (ii) by almost completely blocking the ability of cells to resume proliferation.

CSCs are responsible for the genesis and recurrence of gliomas and show heightened resistance to irradiation and chemotherapy.^24^ CSCs and normal NSCs share core signalling pathways but also display critical distinctions that provide important clues to useful therapeutic targets. In this respect, one therapeutic strategy specifically directed towards the CSC pool consists of forcing these cells to undergo differentiation. For example, glioma CSCs can be strongly driven toward glial or neuronal differentiation by the use of cannabinoid ligands^7^ or inductors of cell autophagy.^56, 57^

In addition to differentiating agents, several inhibitors of neurosphere proliferation have been evaluated.^58^ Appropriate neurotransmission signalling appears to be required for the maintenance of NSCs, and compounds that affect purinergic receptors, among others, affected the formation of spheres of tumour cells.^58^ Therefore, in this study, we focused on the purinergic receptors for adenosine. Each of ARs has indeed a pivotal role in the control of tumour growth and invasiveness^30, 31, 32, 33, 34^ but no data on their role in CSC biology are available to date. We showed that in the CSCs derived from GBM cells, selective AR agonists generally reduced the level of cell proliferation/viability after 4–7 days of treatment. The strongest inhibition of cell proliferation was obtained upon stimulation of the A_1_AR and A_2B_AR subtypes.

Then, the morphology and stemness/differentiation of CSCs upon treatment with selective AR agonists were evaluated. BAY606583 caused a reduction in the area occupied by and the number of CSC spheres, and induced a significant level of CSC apoptosis beginning at 4 days of treatment. However, the effects elicited by the A_2B_AR agonist were reversible and not long lasting. In contrast, CHA-treated cells showed the prominent outgrowth of cellular processes, with a significant reduction in the levels of the stemness markers and a concomitant increase in the levels of the glial markers. As the A_1_AR agonist induced CSC apoptosis after 7 days of treatment but not at the 4th day, we speculated that CHA most likely favours the differentiation of CSCs and the subsequent apoptosis of the differentiated cells. At the same time points, A_1_AR stimulation did not have a significant effect on the proliferation of GBM cells, suggesting that CHA preferentially depleted the differentiated CSCs rather than GBM cells.

Then, the signalling pathways most likely to be implicated in the CHA- or BAY606583-mediated effects on the proliferation and differentiation of CSCs were investigated. It has been demonstrated that the PI3K/AKT/mTOR signalling pathway is critical for the maintenance of the properties of glioma CSCs^59^ and that the dual inhibition of PI3K/AKT/mTOR and MEK/ERK signalling induced the differentiation of and inhibited the tumourigenic potential of CSCs.^44^ In this study, we demonstrated that both of the tested AR agonists inhibited the phosphorylation of both ERK1/2 and AKT, but with different kinetic patterns, suggesting that the kinetics of AKT inhibition may explain the differential effects elicited by the two AR agonists.

The two classes of agonists also had different effects on some aspects of HIF transcriptional activity. Both AR agonists decreased the level of HIF-1*α* transcription at 7 days of treatment, consistent with the data in literature, showing that silencing the HIF-1*α* gene resulted in the inhibition of GBM tumour growth, by both inhibiting the rate of tumour cell migration/invasion^50^ and inducting CSC differentiation.^51, 52^ The A_2B_AR ligand did not affect the level of HIF-2*α* mRNA at the time points used in this study. In contrast, CHA caused a reduction in the level of HIF-2*α* expression after 4 days of treatment, and an increase in its level after 7 days of treatment. The initial decrease in HIF-2*α* expression is consistent with the differentiating properties of A_1_AR; HIF-2*α* has been found to be colocalised with stem cell markers in tumour specimens^45^ and to have a pivotal role in maintaining stem cells in an undifferentiated state.^60^ In contrast, the increase in the HIF-2*α* mRNA levels that was observed on the 7th day may be associated with effects on other signalling pathways in the CHA-differentiated CSCs. For example, the increase in HIF-2*α* expression that was observed at the latter time point may impair Notch signalling activity,^61^ thus inhibiting cell proliferation and favouring a differentiated cell phenotype.^44, 62^

Finally, whether the AR agonists modulated the expression of pro-apoptotic proteins in CSCs was investigated. BAY606583 significantly increased the Bax mRNA levels starting at 4 days of treatment, with a maximal effect at the 7th day. In contrast, CHA increased Bax transcriptional activity only at the latter time point. These results are consistent with the results obtained in the apoptosis analysis and confirmed that whereas the A_2B_AR agonist mainly exerted a pro-apoptotic effect, CHA first induced CSC differentiation and, in a later phase, triggered the apoptosis of the differentiated cells.

We finally examined the effect of combined treatment with TMZ and the A_1_AR or A_2B_AR ligands on the fate of CSCs. We demonstrated that when the cells were treated with TMZ and BAY606583 or CHA in combination, a synergic/additive effect on the inhibition of CSC proliferation and a significant decrease in the ability of treated cells to resume proliferation was observed. Interestingly, in the presence of TMZ, CHA did not induce CSC differentiation, suggesting that TMZ has a dominant role in driving the cellular fate of CSCs, promoting their apoptosis/death rather than their differentiation.

The most interesting results were obtained in the sequential treatment experiments; challenging cells with the AR agonists before treating them with TMZ potentiated the anti-proliferative effects of this chemotherapeutic agent, prolonging its effective period.

These results strongly suggested that modulating the activity of ARs may be an efficient strategy to sensitise glioma CSCs to chemotherapy and that ARs may be invaluable targets in glioma stem cell-orientated therapies.

## Materials and Methods

### Materials

N^6^-cyclo-hexyladenosine (CHA), 3-[4-[2-[[6-amino-9-[(2R,3R,4S,5S)-5-(ethylcarbamoyl)-3,4-dihydroxy-oxolan-2-yl]purin-2-yl]amino]ethyl]phenyl]propanoic acid (CGS21680), 2-chloro-*N*6-(3-iodobenzyl)adenosine-5′-*N*-methylcarboxamide (Cl-IB-MECA), 1,3-dipropyl-8-cyclopentylxanthine (DPCPX) and TMZ were purchased from Sigma-Aldrich (Milan, Italy). [2-[6-Amino-3,5-dicyano-4-[4-(cyclopropylmethoxy)-phenyl]pyridin-2-ylsulfanyl]acetamide] (BAY606583) and MRS1754 were purchased from Tocris Bioscience (Bristol, UK); the 3-(4,5-dimethylthiazol-2-yl)-5-(3-carboxymethoxyphenyl)-2-(4-sulfophenyl)-2H-tetrazolium (MTS) assay kit was obtained from Promega (Milan, Italy). The RNeasy Mini Kit was purchased from Qiagen (Milan, Italy) and the ProtoScript cDNA Synthesis Kit was obtained from Biolabs, Euroclone (Milan, Italy). All of the other reagents that were used were obtained from commercial sources.

### GBM cell line culture and CSC isolation

The U87MG and U343MG cell lines were obtained from the National Institute for Cancer Research of Genoa (Italy) and the Cell Lines Service GmbH (Germany), respectively. The DNA profile of each cell line was monitored. The U87MG and U343MG cells were cultured in RPMI medium and Eagle's minimum essential medium adjusted to contain a final concentration of 1.5 g/l sodium bicarbonate, respectively, supplemented with 10% FBS, 2 mM L-glutamine, 100 U/ml penicillin, 100 mg/ml streptomycin and 1% non-essential amino acids at 37 °C in 5% CO_2_. Both cell lines were sub-cultured when the monolayer reached 75 to 85% confluence, at which point the average cell density was 1.5 × 10^5^ viable cells/cm^2^. To isolate CSCs, approximately 2.0 × 10^6^ cells of each GBM cell line was suspended in 1 ml of a defined serum-free NSC medium containing 20 ng/ml of basic fibroblast growth factor (Sigma-Aldrich), 20 ng/ml of epidermal growth factor (Sigma-Aldrich) and 20 ng/ml or 20 *μ*l/ml of B27 supplement (Life Technologies, Milan, Italy). After 3–4 days of culture, the neurospheres were collected, suspended in NSC medium, dissociated into single cells and plated for the assays. For the long-term treatment of cells, NSC or complete medium containing drugs was replaced every 2–3 days.

### Cell proliferation/viability assays of GBM cells and CSCs

The human GBM cells or CSCs were seeded at a density of 3 × 10^3^ cells per well. After 24 h, the culture medium was replaced with fresh stem cell culture medium, and the cells were treated for 1–7 days with the AR agonists CHA (A_1A_R), CGS21680 (A_2A_AR), BAY606583 (A_2B_AR) and Cl-IB-MECA (A_3_AR) at different concentrations, and with TMZ, alone or in combination. Following the treatment period, cell proliferation was using the MTS assay according to manufacturer's instructions. In some experiments, the effects of the AR antagonists on agonist-mediated effects were evaluated. In particular, the A_1A_R antagonist DPCPX (50 nM) and the A_2B_AR antagonist MRS1754 (20 nM) were used. The antagonists were added 10 min before the addition of 100 nM CHA or 50 nM BAY606583, respectively.

For the drug wash-out experiments, neurospheres were treated for 7 days with 100 nM CHA, 50 nM BAY606583, and 100 *μ*M TMZ, alone or in combination. For sequential treatments, the cells were pre-treated with AR agonists for 7 days and then with TMZ for another 7 days. At the end of treatment periods, the drug-containing media were replaced with fresh drug-free NSC medium, and the cells were allowed to grow for another 14 days.

Within an experiment, each condition was assayed in triplicate, and each experiment was performed at least three times. The results were calculated by subtracting the mean background values from the values obtained under each test condition and were expressed as the percentages of the control (untreated cells) values.

The effects of the combined treatments on CSC viability were also evaluated using the Trypan blue exclusion assay. U87MG-derived CSCs were treated for 1–7 days with CHA, CGS21680, BAY606583 and Cl-IBMECA at the indicated concentrations and with TMZ (100 *μ*M), alone or in combination. Following the treatment period, cells were collected and centrifuged at 300 × *g* for 5 min. The harvested cells were mixed with an equal volume of 0.4% Trypan blue dye, and the blue (dead cells) and white (living cells) cells in each well were manually counted. The number of live cells for each condition was reported as the percentage of living cells relative to that in the control sample.

### [^35^S]GTPγS-binding assay in CSCs

[^35^S]GTP*γ*S binding to membranes obtained from CSCs was assayed as previously described, with some modifications.^63^ Briefly, cell membranes (30 *μ*g) obtained from U343MG-derived CSCs were incubated in the assay buffer (50 mM Tris-HCl, 1 mM EDTA, 1 mM MgCl_2_, 100 mM NaCl, 1 mM DL-dithiothreitol, 0.0005% Tween 20 and 0.5% bovine serum albumin) in the presence of 2 units/ml adenosine deaminase, 1 *μ*M GDP and the AR agonists at different concentrations. Binding was initiated by the addition of 0.2 nM [^35^S]GTP*γ*S and the incubation was continued for 30 min at 25 °C. The level of nonspecific binding was determined using 10 *μ*M unlabelled GTP*γ*S. The reaction was stopped by rapid filtration and the plates were washed twice using 200 *μ*l of buffer. The levels of radioactivity were measured using liquid scintillation spectrometry.

### Western blot analysis

CSCs were treated with DMSO (control), 100 nM CHA or 50 nM BAY606583 for 7 days. At the end of the treatment period, the cells were collected and then were lysed for 60 min at 4 °C using 200 *μ*l of RIPA buffer (9.1 mM NaH_2_PO_4_, 1.7 mM Na_2_HPO_4_, 150 mM NaCl, pH 7.4, 0.5% sodium deoxycholate, 1% Nonidet P-40, 0.1% SDS and a protease-inhibitor cocktail). Equal amounts of the cell extracts (40 *μ*g of protein) were diluted in Laemmli sample solution, resolved using SDS-PAGE (8.5%), transferred to PVDF membranes and probed overnight at 4 °C using the following primary antibodies: anti-nestin (sc-20978, Santa Cruz Biotechnology, Heidelberg, Germany; 1:50); anti-GFAP (sc-9065, Santa Cruz Biotechnology; 1:50); anti-A_1A_R (sc-19223, Santa Cruz Biotechnology; 1:200), anti-A_2A_AR (sc-13937, Santa Cruz Biotechnology; 1:200); anti-A_2B_AR (sc-28996, Santa Cruz Biotechnology; 1:50); anti-A_3_AR (sc-13938, Santa Cruz Biotechnology; 1:200); and anti-caspase-3 (sc-7148, Santa Cruz Biotechnology; 1:100). The primary antibodies were detected using the appropriate peroxidase-conjugated secondary antibodies, which were then detected using a chemioluminescent substrate (ECL, Perkin Elmer, Waltham, MA, USA). Densitometric analysis of the immunoreactive bands was performed using ImageJ Software (version 1.41; Bethesda, MD, USA).

### Quantitation of the occupied area and the cellular processes of neurospheres

CSCs isolated from U343MG cells were plated in NSC medium (day 0) and treated for 7 days with 100 nM CHA, 50 nM BAY606583, and 100 *μ*M TMZ, alone or in combination (until day 7). In some experiments, the CSCs were treated with the indicated AR agonist (until day 7), and subsequently with TMZ for another 7 days (until day 14). At the end of the treatment periods, the drug-containing media were replaced with fresh NSC medium, and the CSCs were allowed to grow for another 7 or 14 days. Photographs of the neurospheres were taken at days 0, 7, 14 and 21; three different wells were analysed for each condition, 15 images of each well were captured (using a 20 × objective lens). The response of the cultures to the various treatments was quantified by measuring the area occupied by neurospheres that had formed, using the ImageJ program. The cellular processes extending from the six to eight differentiating neurospheres per condition in three independent experiments were evaluated. To measure the cellular processes, the average diameter of an individual neurosphere in the vertical and horizontal planes was determined. The number of extensions from the body of the neurospheres was counted, and the ratio of the number of extensions to the average neurosphere diameter was calculated.^64^ In addition, the neurite lengths were measured. All of the measurements were performed using the ImageJ Program (http://rsbweb.nih.gov/ij/docs/faqs.html#cite).

### RNA extraction and real-time PCR analysis

CSCs were treated with proliferation medium containing DMSO (control), CHA, CGS21680, BAY606583 or Cl-IBMECA at the indicated concentrations or TMZ for 4 or 7 days. At the end of the treatment period, the cells were collected, and total RNA was extracted using RNeasy Mini Kit (Qiagen, Hilden, Germany) according to manufacturer's instructions. The purity of the RNA samples was evaluated by measuring the absorbance at 260 and 280 nm. cDNA synthesis was performed with 500 ng of RNA using the i-Script cDNA synthesis kit (Bio-Rad, Hercules, CA, USA) according to the manufacturer's instructions. The primers used for RT-PCR were designed to anneal to the intron/exon boundaries to ensure that the amplified products did not include genomic DNA. The RT-PCR reaction mixtures consisted of 25 *μ*l of Fluocycle II SYBR (Euroclone), 1.5 *μ*l of 10 *μ*M forward and reverse primers, 3 *μ*l of cDNA, and 19 *μ*l of H_2_O. All of the reactions were performed for 40 cycles using the following temperature profile: 98 °C for 30 s (initial denaturation); T °C (see Supplementary Table 1) for 30 s (annealing); and 72 °C for 3 s (extension). *β*-Actin was used as the housekeeping gene. The levels of mRNA in each sample were normalised against the level of *β*-actin mRNA, and the relative expression was calculated using the Ct value. The specificity of the assays was determined by both melting curve analysis and gel electrophoresis, and the data were analysed using the standard-curve method. For the quantitation of AR expression in CSCs, the data were expressed as the fold change relative to the level of expression of each AR subtype in whole GBM cells.

### Annexin V and 7-AAD staining

Dual staining with Annexin V conjugated to fluorescein-isothiocyanate (FITC) and 7-amino-actinomysin (7-AAD) was performed using a commercially available kit (Muse Annexin V and Dead Cell Kit; Merck KGaA, Darmstadt, Germany). Briefly, CSCs were treated with DMSO (control), 100 nM CHA, 500 nM CGS21680, 50 nM BAY606583, 5 nM Cl-IBMECA or 100 *μ*M TMZ, alone or in combination, for 4 or 7 days. Both the floating and adherent cells were collected, centrifuged at 300 × *g* for 5 min and suspended in cell culture medium. Then, a 100 *μ*l aliquot of the cell suspension (approximately 5 × 10^4^ cell/ml) was added to 100 *μ*l of fluorescent reagent and incubated for 10 min at room temperature. Subsequently, the percentages of living, apoptotic and dead cells were determined using a Muse Cell Analyzer in accordance to the manufacturer's guidelines. In cells undergoing apoptosis, Annexin V binds to phosphatidylserine, which is translocated from the inner to the outer leaflet of the cytoplasmic membrane. Double staining was used to distinguish the viable, early-stage apoptotic, and necrotic or late-stage apoptotic cells. Annexin V–FITC-positive and/7-AAD-positive cells were identified as in the early apoptotic stage. Cells that were Annexin V–FITC positive and 7-AAD positive were identified as cells in late-stage apoptosis or as necrotic cells, respectively.

### ERK and AKT phosphorylation assays

CSCs isolated from U87MG cells were collected and were treated for 5 or 30 min with DMSO, 100 nM CHA or 50 nM BAY606583. At the end of the treatment period, the CSCs were centrifuged at 500 × *g* for 3 min, washed twice using fresh saline, and then were rapidly fixed using 8% formaldehyde to preserve specific modified proteins in the activated state. The levels of total and phosphorylated AKT and ERK1/2 were determined using Fast Activated Cell-Based ELISA Kits (Active Motif, Carlsbad, CA, USA), using specific primary antibodies. The subsequent incubation with a secondary HRP-conjugated antibody and the developing solution allowed the colorimetric quantification of the levels of total and phosphorylated proteins. The relative number of cells in each well was then determined using the Crystal-Violet assay. The results were calculated by subtracting the mean background value from the values obtained under each test condition: values were normalised to the number of cells in each well and were expressed as the percentages of the control (untreated cells) values.

### Statistical analyses

The nonlinear multipurpose curve-fitting program Graph-Pad Prism (GraphPad Software Inc., San Diego, CA, USA) was used to analyse the data and prepare the graphic presentations. All of the data were expressed as the mean values±S.E.M. The data were analysed using a one-way analysis of variance (ANOVA) with Bonferroni's corrected *t*-test for post-hoc pair-wise comparisons. *P*<0.05 was considered statistically significant.

## Abbreviations

GBM: glioblastoma multiforme
CSCs: cancer stem cells
ARs: adenosine receptors
TMZ: temozolomide
