# Supplementary figures and images for: Modulation of A1 and A2B adenosine receptor activity: a new strategy to sensitise glioblastoma stem cells to chemotherapy
Source: Cell Death Dis. 2014 Nov 27;5(11):e1539–. doi: 10.1038/cddis.2014.487 (PMC4260745; doi:10.1038/cddis.2014.487)

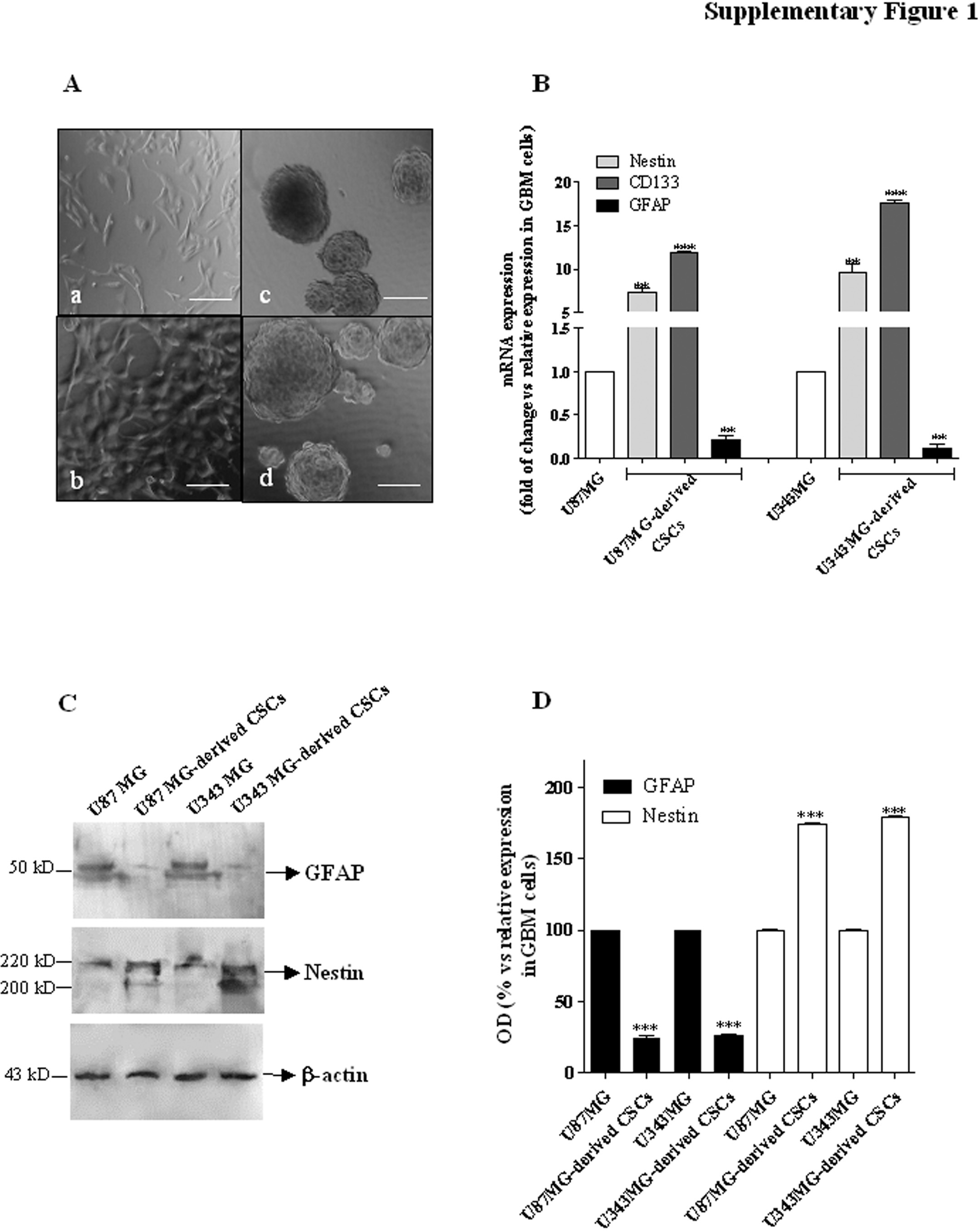

Supplement: Supplementary Figure 1 [file cddis2014487x1.tif]

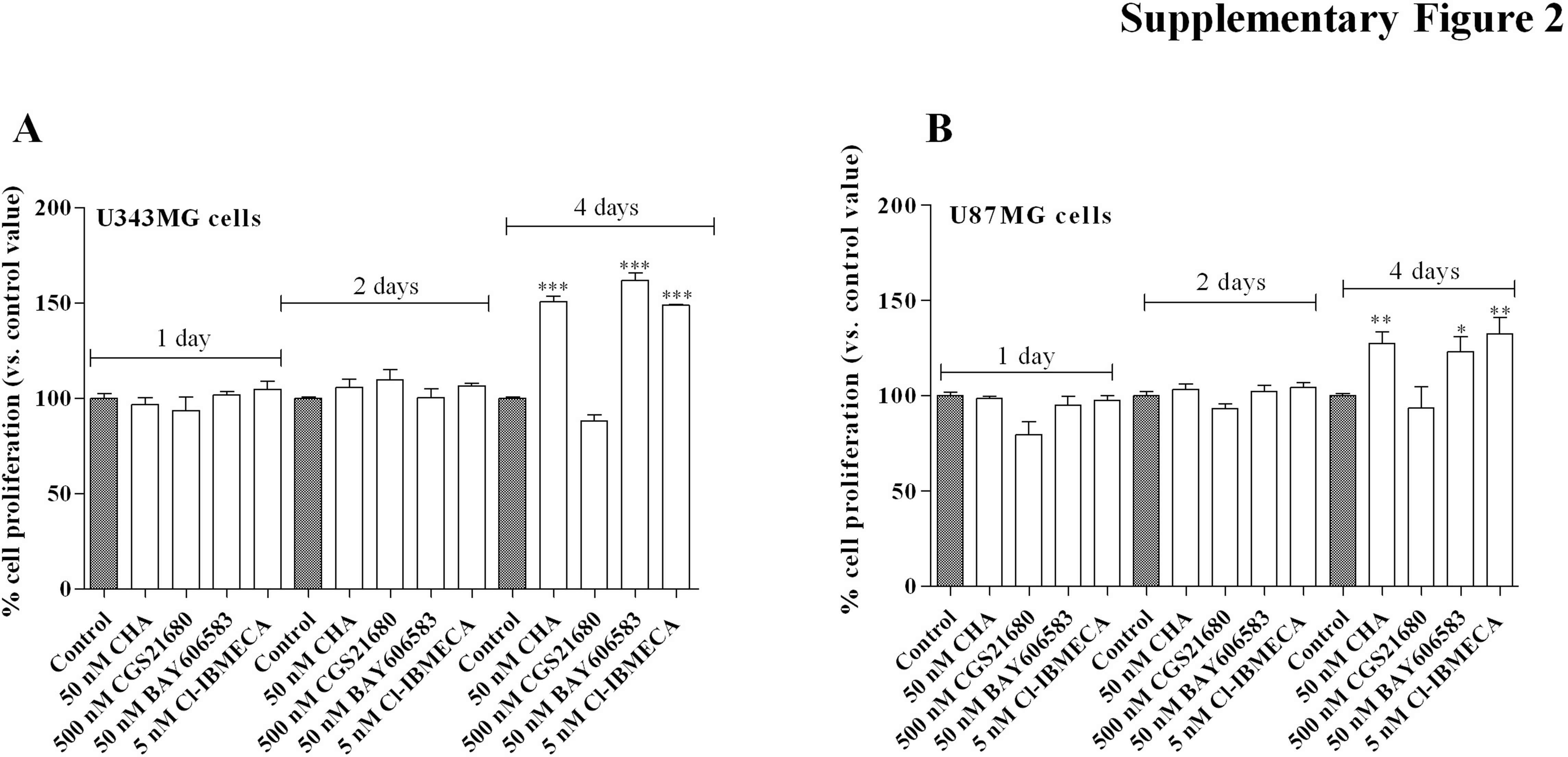

Supplement: Supplementary Figure 2 [file cddis2014487x2.tif]

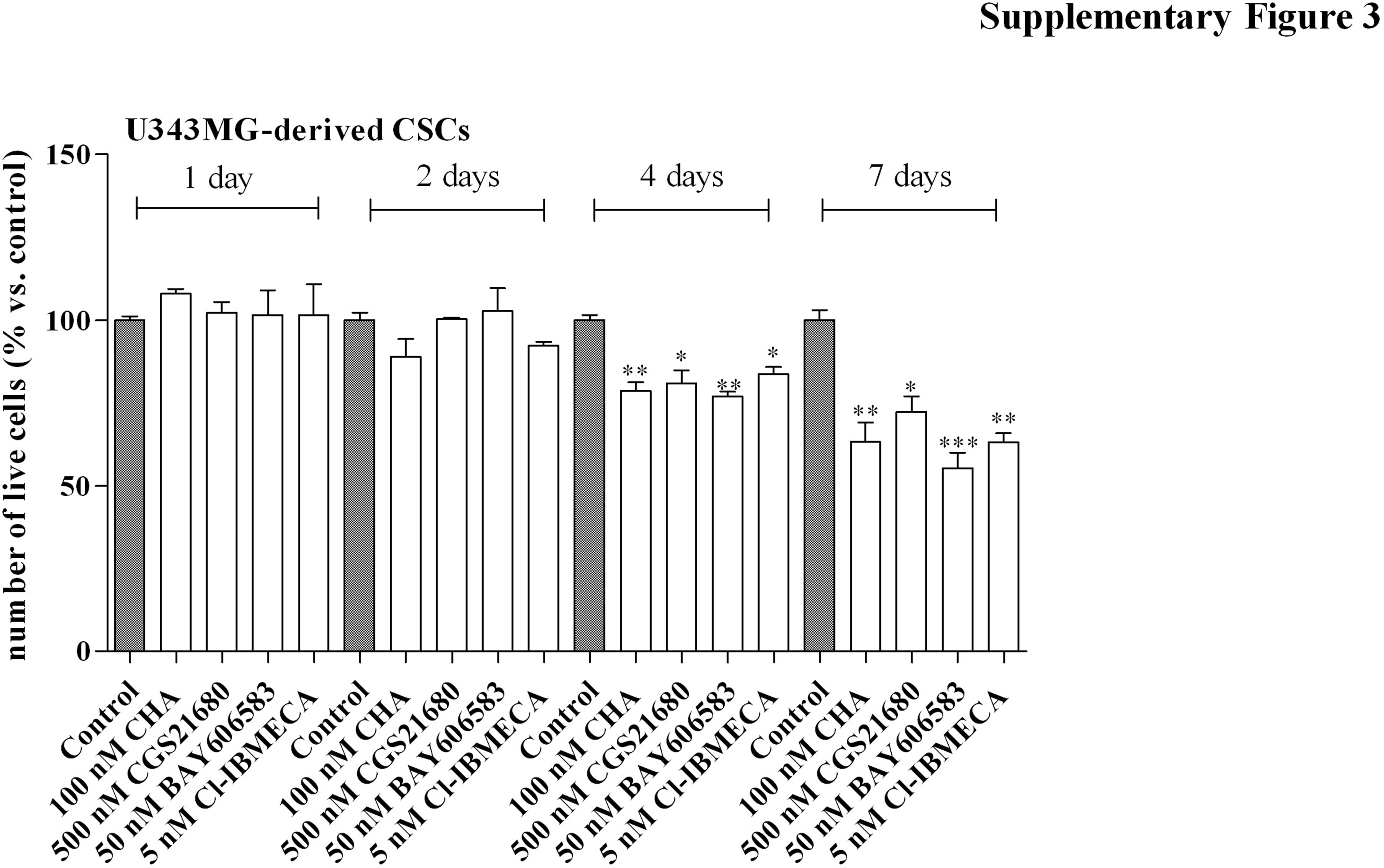

Supplement: Supplementary Figure 3 [file cddis2014487x3.tif]

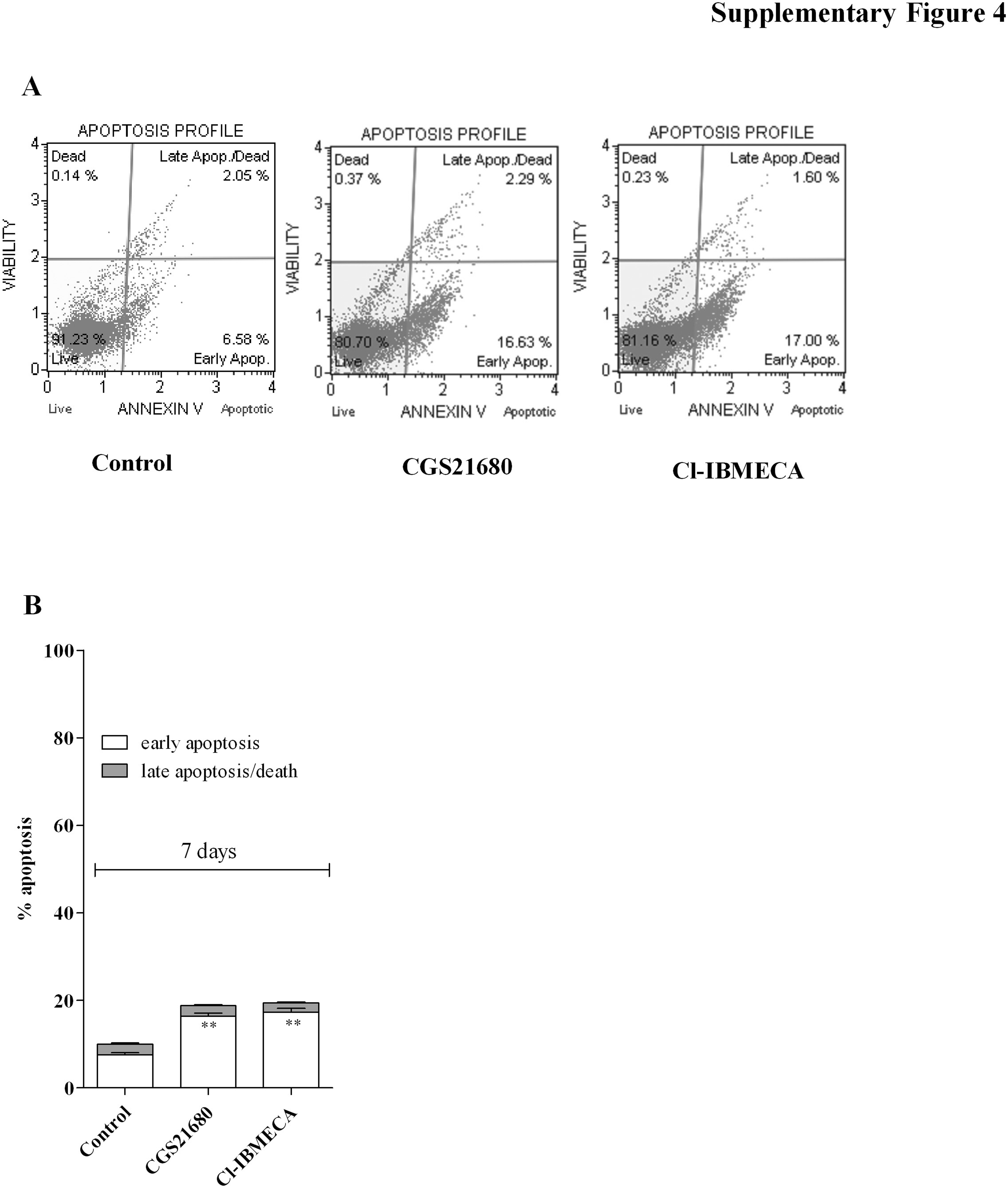

Supplement: Supplementary Figure 4 [file cddis2014487x4.tif]

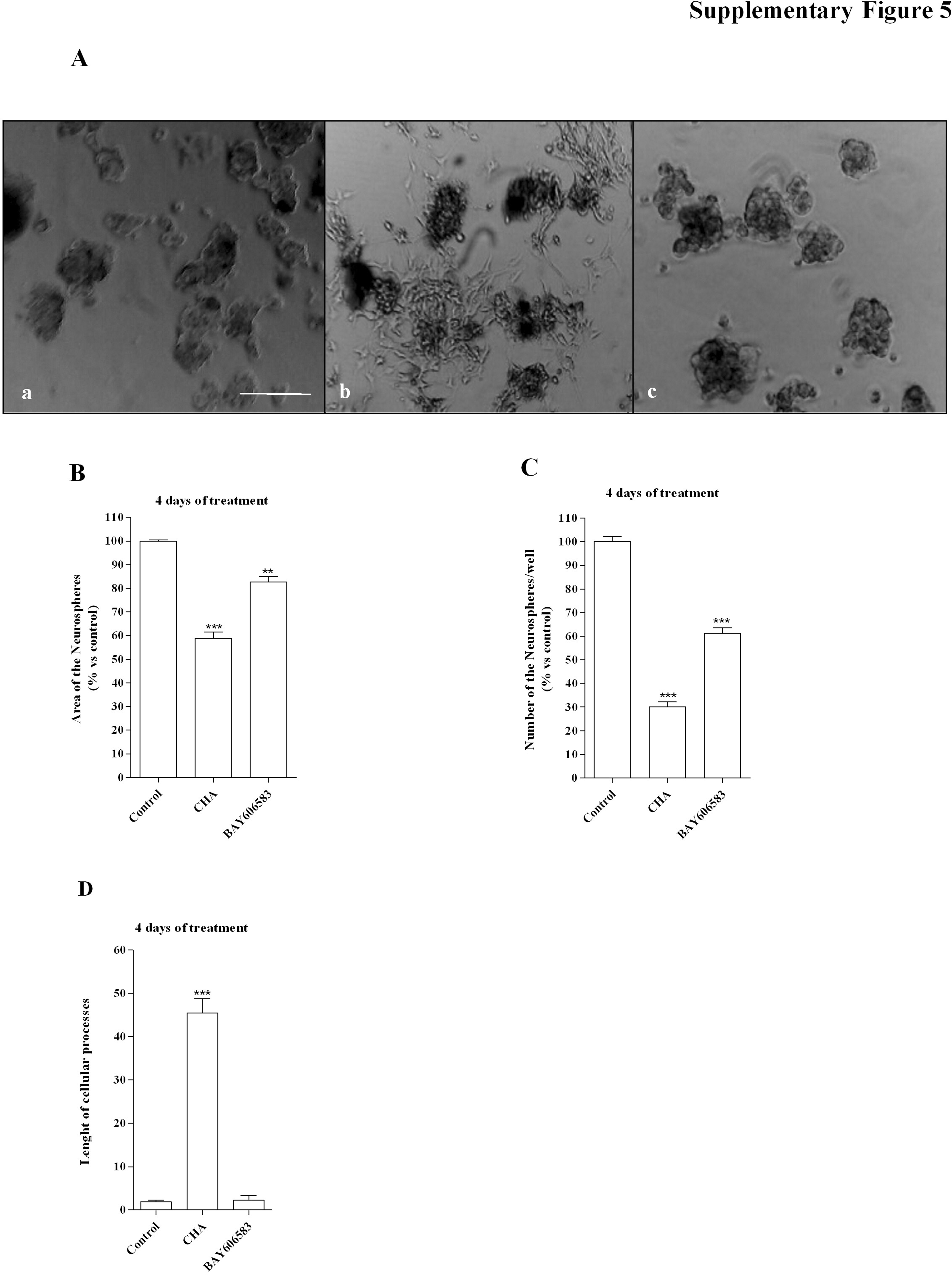

Supplement: Supplementary Figure 5 [file cddis2014487x5.tif]

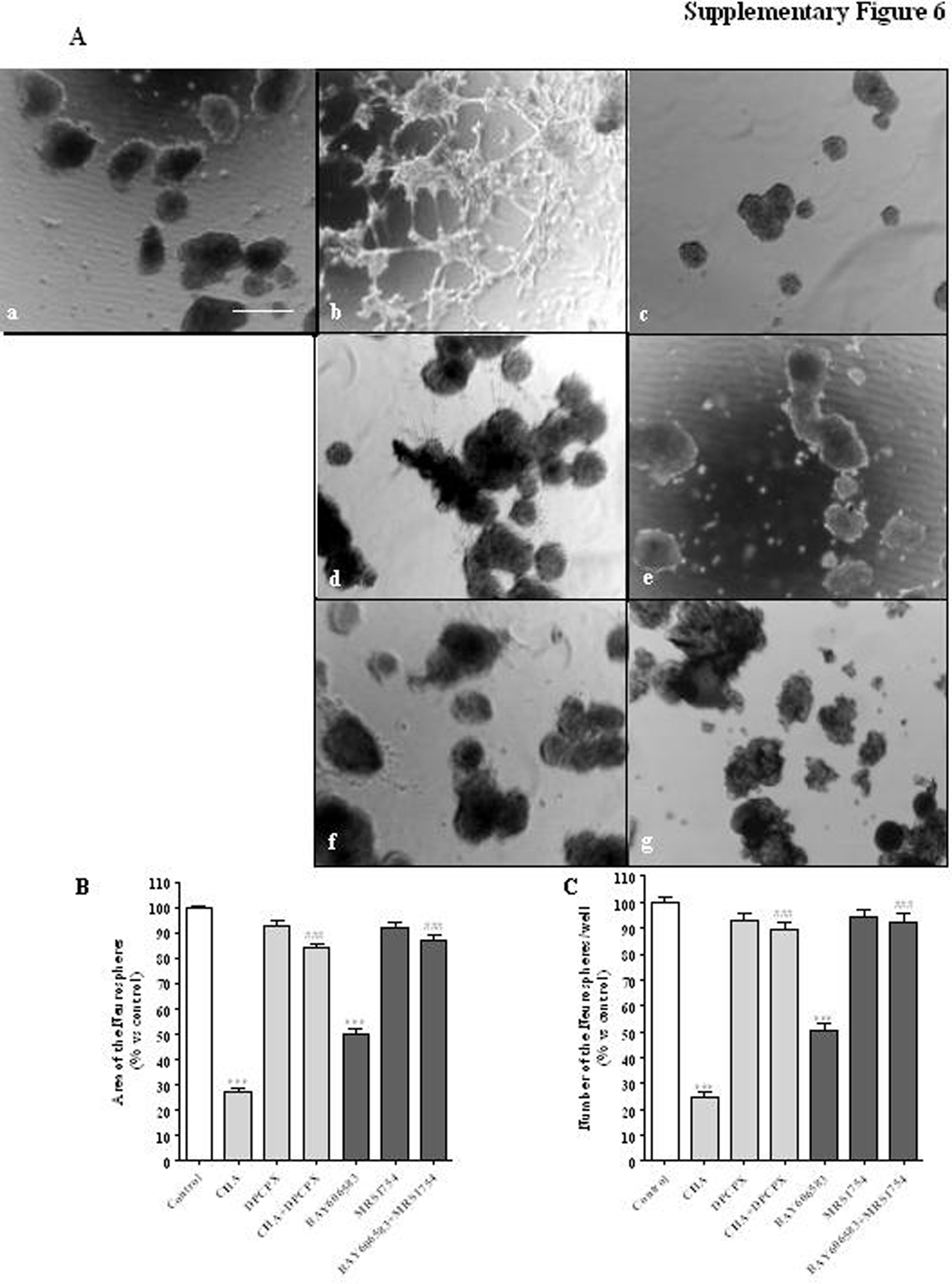

Supplement: Supplementary Figure 6 [file cddis2014487x6.tif]

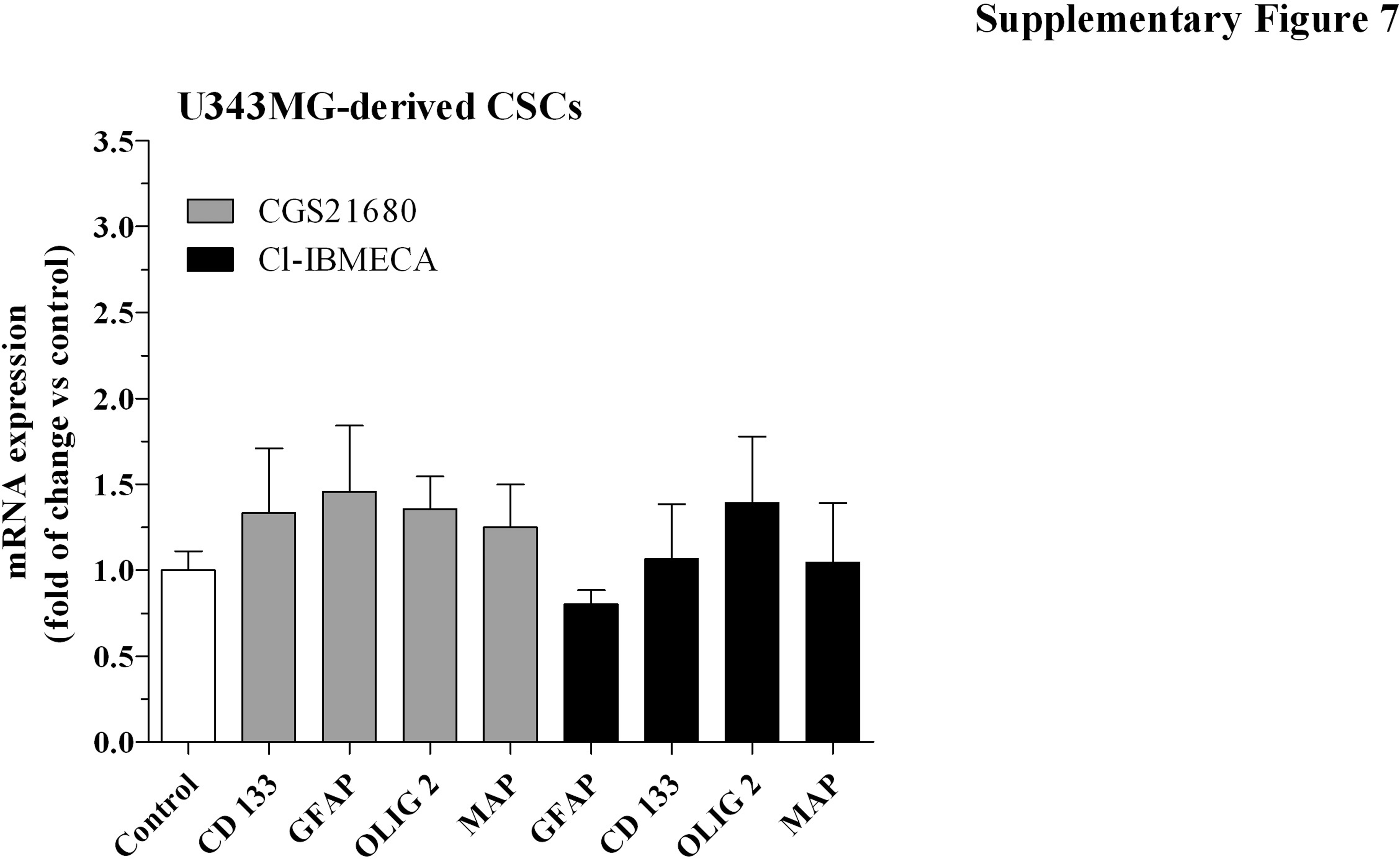

Supplement: Supplementary Figure 7 [file cddis2014487x7.tif]

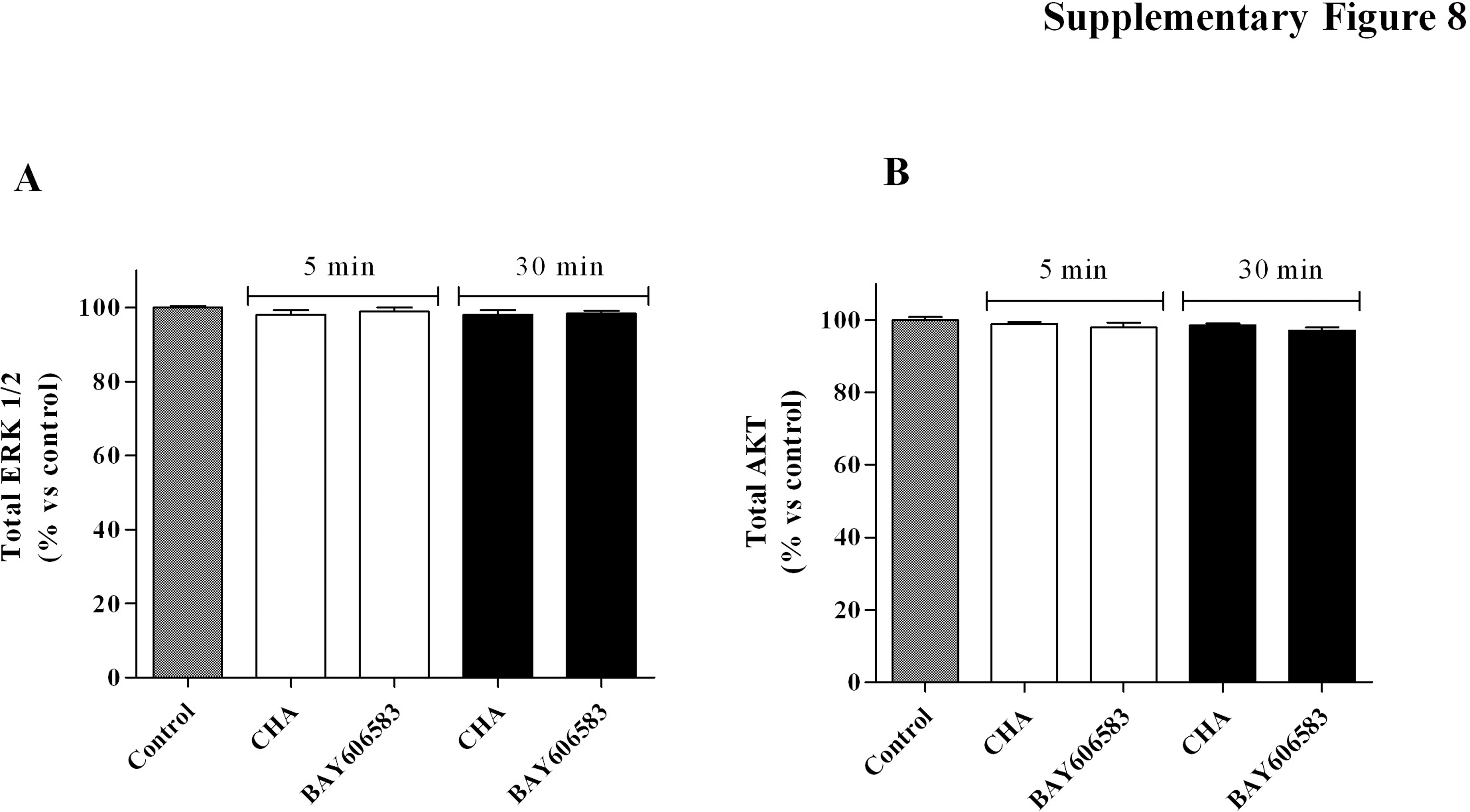

Supplement: Supplementary Figure 8 [file cddis2014487x8.tif]

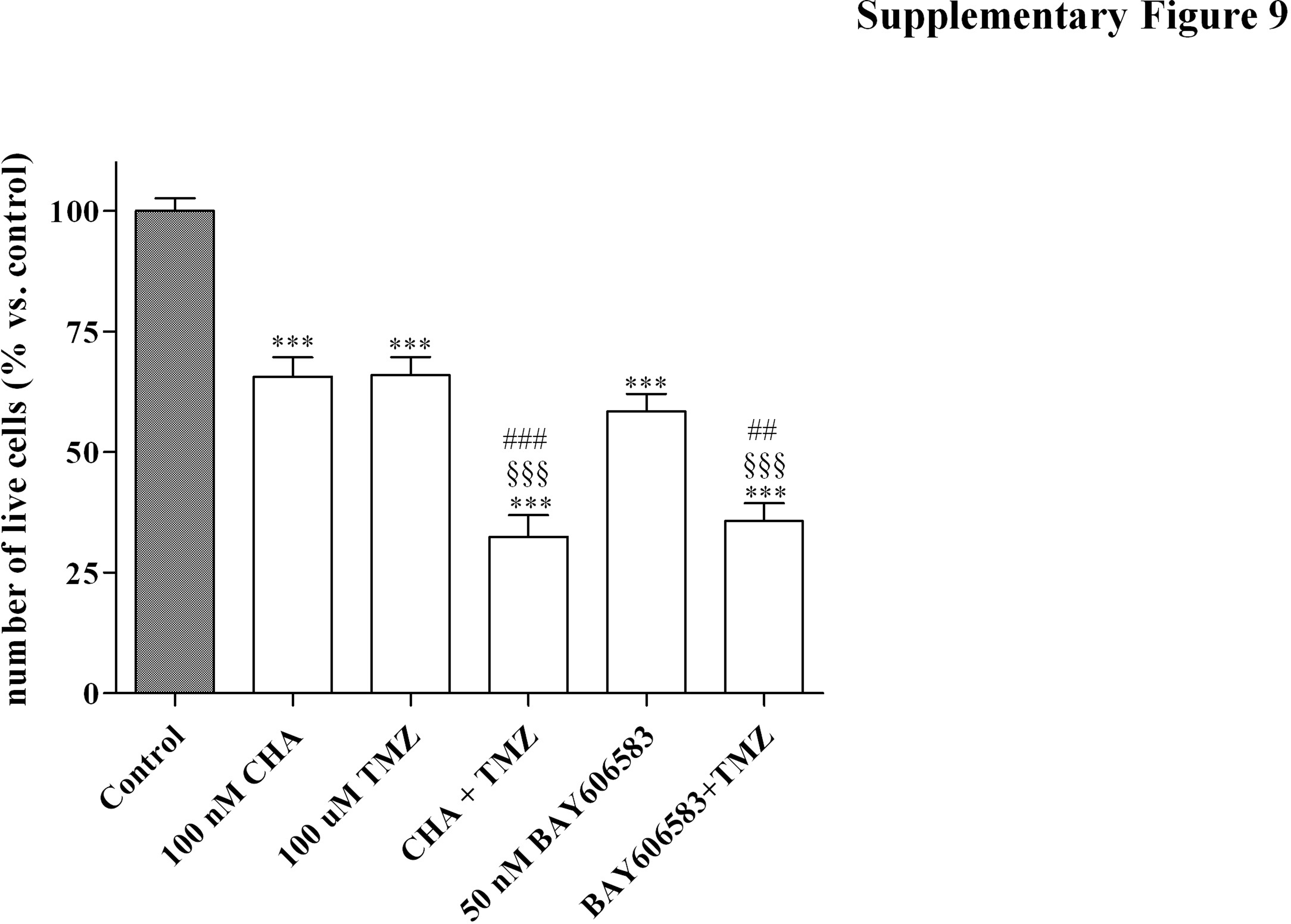

Supplement: Supplementary Figure 9 [file cddis2014487x9.tif]
