# Supplementary material for: Modulation of A1 and A2B adenosine receptor activity: a new strategy to sensitise glioblastoma stem cells to chemotherapy
Source: Cell Death Dis. 2014 Nov 27;5(11):e1539–. doi: 10.1038/cddis.2014.487 (PMC4260745; doi:10.1038/cddis.2014.487)
Supplement: Supplementary Information [file cddis2014487x10.doc]

**Modulation of A_1_ and A_2B_ Adenosine Receptor Activity: a new strategy to sensitise glioblastoma stem cells to chemotherapy**

**Running Title: Adenosine receptors in glioma cancer stem cells**

Simona Daniele^1^, Elisa Zappelli^1,2^, Letizia Natali^1^, Claudia Martini^1,*^, Maria Letizia Trincavelli^1^.

^1^Department of Pharmacy, University of Pisa, 56126 Pisa, Italy.

*Corresponding Author: Department of Pharmacy, University of Pisa, Via Bonanno, 6, Pisa 56126, Italy. Tel.: +390502219522-509; FAX: +390502219609; e-mail address: claudia.[martini@unipi.it](mailto:martini@unipi.it)

^2^These authors contributed equally to this work.

**Supplementary Figure Legends**

**Suppl. Figure 1. Characterization of CSCs derived from U87MG and U343MG cells. A)** Representative bright field microscope images showing whole U87MG (*a*), whole U343MG (*b*), and the respective derived CSCs (*c*, *d*). **B)** The total RNA was extracted from U87MG cells, U343MG cells and from the respective derived CSCs. The relative mRNA quantification of the stem cell markers (CD133 and Nestin) and of the astrocyte marker GFAP was performed by real-time PCR as described in the Methods section. The data were expressed as the fold change relative to the level of expression in GBM cells (U87MG and U343MG), and they are the mean values ± SEM of three different experiments. **C, D)** Cell lysates were prepared from U87MG cells, U343MG cells, and the derived CSCs, and then GFAP and nestin protein levels were evaluated by Western blot analysis. β-actin was used as the loading control. C) Representative Western blots. D) Densitometric analysis of the immunoreactive bands performed using ImageJ program. The data were expressed as the percentage relative to the level of expression in whole GBM cells, and they are the mean values ± SEM of three different experiments. Statistical significance was determined with a one-way ANOVA with Bonferroni post-test: **P<0.01, ***P<0.001 vs the relative expression in GBM cells.

**Suppl. Figure 2. Effects of AR agonists on GBM proliferation.** U343MG (**A**) or U87MG (**B**) were incubated for the indicated number of days with the A_1_AR agonist (CHA), or the A_2A_AR agonist (CGS21680), or the A_2B_AR agonist (BAY606583), or the A_3_AR agonist (Cl-IBMECA) at selected concentration (corresponding to ten fold the affinity constant values). At the end of treatments, cell proliferation was evaluated using the MTS assay. The data were expressed as a percentage with respect to that of untreated cells (control), set to 100%, and are the mean values ± SEM of three independent experiments, each performed in duplicate. Statistical significance was determined with a one-way ANOVA with Bonferroni post-test: *P<0.05, **P<0.01, ***P<0.001 vs control.

**Suppl. Figure 3. Effects of AR agonists on CSC viability.** U343MG-derived CSCs were incubated for the indicated number of days with a selected concentration of CHA, CGS21680, BAY606583, or Cl-IBMECA. At the end of the treatments, living and dead cells were estimated using the trypan blue exclusion test. The data were expressed as a percentage of living cell number for well, and are the mean values ± SEM of three independent experiments, each performed in duplicate. Statistical significance was determined with a one-way ANOVA with Bonferroni post-test: *P<0.05, **P<0.01, ***P<0.001 vs control.

**Suppl. Figure 4. Effects of A_2A_AR and A_3_AR agonists on CSC apoptosis. A, B)** U343MG-derived CSCs were treated for 7 days with NSC medium containing DMSO (control), or 500 nM CGS21680, or 5 nM Cl-IBMECA. At the end of the treatments, the cells were collected and the degree of phosphatydilserine externalization was evaluated using the annexin V protocol as described in the Methods section. B) The data are expressed as the percentage of apoptotic cells (Early-apoptotic in white, late-apoptotic/necrotic in grey) relative to the total number of cells. The data shown are the mean values ± SEM of three different experiments. Statistical significance was determined with a one-way ANOVA with Bonferroni post-test: **P<0.01 vs control.

**Suppl. Figure 5. Effect of A_1_AR and A_2B_AR agonists on sphere-derived cell morphology. A)** CSCs were treated for 4 days with complete NSC medium containing DMSO (control, *a*), or 100 nM CHA (*b*), or 50 nM BAY606583 (*c*). Representative cell micrographs are shown. The area of the culture plates occupied by the spheres (**B**), the number of spheres (**C**), and the length of cellular processes (**D**) were scored after 4 days of treatment. The counts represent the mean values ± SEM of three independent experiments. Statistical significance was determined with a one-way ANOVA with Bonferroni post-test: **P<0.01, ***P<0.001 vs control.

**Suppl. Figure 6. Effect of A_1_AR and A_2B_AR antagonists on sphere-derived cell morphology.** CSCs were treated for 7 days with complete NSC medium containing DMSO (control, *a*), 100 nM CHA (*b*), 50 nM BAY606583 (*c*), the A_1_AR antagonist DPCPX (50 nM, *d*), the A_2B_AR antagonist MRS1754 (20 nM, *e*), CHA plus DPCPX (*f*), or BAY606583 plus MRS1754 (*g*). The area of the culture plates occupied by the spheres (**B**) and the number of spheres (**C)** were scored after 7 days of treatment. The counts represent the mean values ± SEM of three independent experiments. Statistical significance was determined with a one-way ANOVA with Bonferroni post-test: ***P<0.001 vs control; ###P<0.001 vs agonist alone.

**Suppl. Figure 7. Effects of A_2A_AR and A_3_AR agonists on differentiation and stemness markers in CSCs. A**) CSCs derived from U343MG cells were treated for 7 days with NSC medium containing DMSO (control), or 500 nM CGS21680, or 5 nM Cl-IBMECA. At the end of treatment periods, the total RNA was extracted, and relative mRNA quantification of the mRNAs for the stem cell marker CD133, of the astrocyte marker GFAP, of the neuronal marker MAP and of the oligodendrocyte marker Olig2 were performed using real time PCR as described in the Methods section. The data were expressed as the fold change vs the levels of the control, and they are the mean values ± SEM of three different experiments. Statistical significance was determined with a one-way ANOVA with Bonferroni post-test.

**Suppl. Figure 8. Effects of A_1_AR and A_2B_AR agonists on the levels of total ERK1/2 and AKT.** U343MG-derived CSCs were treated for 5 or 30 min with complete medium containing DMSO (control), or 100 nM CHA, or 50 nM BAY606583. Following the treatments, the levels of total ERK 1/2 (**A**) or AKT (**B**) were evaluated using ELISA kit as described in the Methods section. The data were expressed as the percentage of total AKT or ERK1/2 relative to those of untreated cells (control) set to 100%, and are the mean values ± SEM of three independent experiments performed in triplicate. Statistical significance was determined with a one-way ANOVA with Bonferroni post-test.

# Suppl. Figure 9. Effect of the combination of TMZ and A_1_AR or A_2B_AR agonists on CSC viability. U343MG-derived CSCs were incubated for 7 days with 100 nM CHA, 50 nM BAY606583, 100 µM TMZ, alone or in combination. At the end of treatment periods, living and dead cells were estimated using the trypan blue exclusion test. The data were expressed as the percentage of living cell number for well, and they are the mean values ± SEM of three independent experiments, each performed in duplicate. Statistical significance was determined with a one-way ANOVA with Bonferroni post-test: ***P<0.001 vs control; ##P<0.01, ###P<0.001vs AR agonist alone; §§§P<0.001 vs TMZ alone.

# Suppl. Table 1. Nucleotide sequences, annealing temperature and product size of the primers utilized in Real Time PCR experiments.

| **Gene** | **Primer nucleotide sequences** | **Annealing Temperature**  **(°C)** | **Product size**  **(base pairs)** |
| --- | --- | --- | --- |
| GFAP | FOR: 5’-CCTCTCCCTGGCTCGAATG -3’  REV: 5’-GACAACCGCCACTCAACTAGC-3’ | 52 | 287 |
| CD133 | FOR: 5’-TCCACAGAAATTTACCTACATTGG -3’  REV: 5’-CAGCAGTTCAAGACGCAGATGACCA-3’ | 61 | 251 |
| MAP | FOR: 5’-TTGGTGCCGAGTGAGAAGAA -3’  REV: 5’-GGTCATGCTGGCAGTGGTTGGT -3’ | 55 | 280 |
| OLIG2 | FOR: 5’-CAGAAGCGCTGATGGTCATA -3’  REV: 5’-TCGGCAGTTTTGGGTTATTC -3’ | 55 | 149 |
| NESTIN | FOR: 5’-CAGCGTTGGAACAGAGGTTGG -3’  REV: 5’-TGGCACAGGTGTCTCAAGGG -3’ | 62 | 282 |
| HIF-1α | FOR: 5’-AAAGGACAAGTCACCACAGG -3’  REV: 5’-TTCTGTTTGTTGAAGGGAG -3’ | 50 | 216 |
| HIF-2α | FOR: 5’-AGCCTCCATCTGCCATCAGTC -3’  REV: 5’-CTTGCCATGCCTGACACCTTG -3’ | 50 | 131 |
| BAX | FOR: 5’-TTTGCTTCAGGGTTTCATCC -3’  REV: 5’-CAGTTGAAGTTGCCGTCAGA -3’ | 55 | 219 |
| A_1_AR | FOR: 5’-TCCCTCTCCGGTACAAGATG -3’  REV: 5’-GCTGCTTGCGGATTAGGTAG-3’ | 55 | 300 |
| A_2A_AR | FOR: 5’-TCTTCAGTCTCCTGGCCATC-3’  REV: 5’-TCCAACCTAGCATGGGAGTC-3’ | 55 | 156 |
| A_2B_AR | FOR: 5’-TCCATCTTCAGCCTTCTGGC -3’  REV: 5’-AAAGGCAAGGACCCAGAGGA-3’ | 55 | 128 |
| A_3_AR | FOR: 5’-CAGCAAAGCGTCAACTCGTGC -3’  REV: 5’-CAAACGGGAGAAGCAGAGGAAC-3’ | 55 | 118 |
| β-actin | FOR: 5’-GCACTCTTCCAGCCTTCCTTCC-3’  REV-5’-GAGCCGCCGATCCACACG-3’ | 55 | 254 |
